# Sprayable Cellulose and Mannan Nanocrystals from Ivory Nuts for Treatment of Skin Diseases in Mice

**DOI:** 10.1021/acsabm.5c01708

**Published:** 2025-11-21

**Authors:** Vanessa M. E. da Rocha, Ana Paula B. Wille, Ana Paula S. e Silva, Matheus S. Gularte, Mauro P. Soares, Marcelle M. Silveira, Janice Giongo, Rodrigo A. Vaucher, Jeff R. Spitzner, André R. Fajardo, Enrique Javier Carvajal Barriga, Ethel A. Wilhelm

**Affiliations:** † Preclinical and Translational Research Group in Pain and Chronic Diseases, 37902 Federal University of Pelotas (UFPel), 96010-900 Pelotas, RS, Brazil; ‡ Laboratory of Technology and Development of Composites and Polymer Materials (LaCoPol), Federal University of Pelotas (UFPel), 96010-900 Pelotas, RS, Brazil; § Regional Diagnostic Laboratory Faculty of Veterinary Medicine, Federal University of Pelotas (UFPel), 96010-900 Pelotas, RS, Brazil; ∥ Laboratory of Biochemistry Research and Molecular Biology of Microorganisms (LaPeBBioM), Federal University of Pelotas (UFPel), 96010-900 Pelotas, RS, Brazil; ⊥ Faculty of Medicine, 67820 Federal University of Rio Grande, 96203-900 Rio Grande, RS, Brazil; # Neotropical Center for the Biomass Research (CNIB), 27884 Pontificia Universidad Católica del Ecuador (PUCE), Av. 12 de Octubre 1076 y Roca, Quito 170109, Ecuador; ∇ PanoMatrix, LLC, 1476 Manning Pkwy, Powell, Ohio 43065, United States

**Keywords:** nanocellulose, biomaterials, wound dressing, chronic skin diseases, streptozotocin-induced diabetes, histological analysis

## Abstract

Atopic dermatitis (AD) and diabetic wounds are chronic inflammatory skin conditions with limited treatment options. This study investigates the therapeutic potential of sprayable colloidal suspensions composed of cellulose and mannan nanocrystals (CNC/MN) derived from ivory nuts in preclinical models of AD and diabetic wound healing. AD was induced in BALB/c mice using 2,4-dinitrochlorobenzene (DNCB), while diabetes was induced in Swiss mice via streptozotocin before dorsal wounds were created. AD severity was assessed through clinical scoring, scratching behavior, histopathology, oxidative stress markers, inflammatory profiling, and emotional domain evaluation. Wound closure rates, bacterial burden, and histological analysis were used to evaluate diabetic wound healing. CNC/MN-based suspensions alleviated DNCB-induced inflammatory skin damage (back: around 48%, and dorsal skin: around 78%) and reversed depressive-like behavior (around 50%) without affecting locomotor activity. The formulation with higher MN content showed superior efficacy in reducing erythema, edema, and neutrophilic infiltration while restoring antioxidant enzyme activity. In diabetic wounds, suspensions with lower MN or without MN content exhibited the best results, enhancing wound closure, collagen deposition, and reducing inflammation. The CNC/MN-based suspension with lower MN content significantly reduced bacterial colonization in the wound site (around 23%). These findings demonstrate that CNC/MN colloidal suspensions are promising sprayable biomaterials for treating inflammatory skin disorders, mitigating cutaneous and neuropsychiatric AD symptoms while promoting tissue regeneration in diabetic wounds. This study highlights their dual therapeutic potential and sustainable origin, offering an innovative treatment alternative for chronic skin disease.

## Graphical abstract

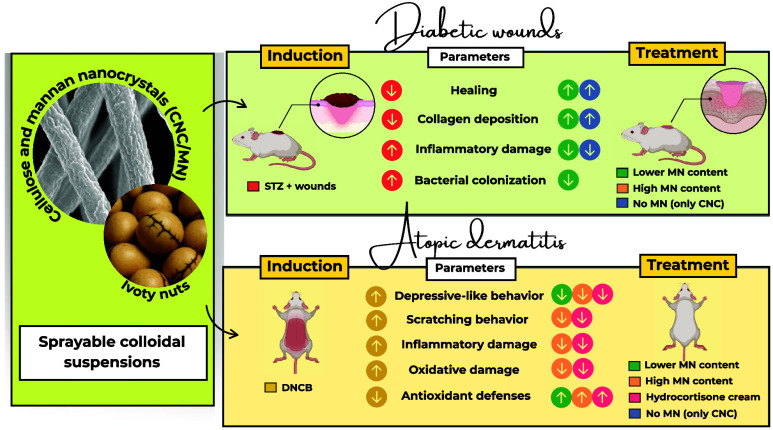

## 1 Introduction

The skin is the human body’s largest organ, serving as a protective barrier against external factors such as chemical substances, environmental conditions, and infectious agents like bacteria and viruses.^1^ Additionally, the skin plays a crucial role in regulating body temperature and sensory perception.^1^ Despite its resilience, the skin’s health and functionality can be significantly compromised by a variety of external and internal factors, including trauma, injuries, diseases, and biological influences such as age and gender.^2^ Among the many skin-related health issues, disorders caused by diseases are particularly concerning due to their wide range of symptoms, severity, and duration. Some skin disorders not only cause physical discomfort but also emotional distress, and in severe cases, they can pose life-threatening risks.

One of the most common chronic skin disorders is atopic dermatitis (AD), which is characterized by extreme itching, inflammation, irritation, and redness of the skin.^3^ The severity of AD symptoms can vary significantly between individuals; in some cases, repeated scratching can lead to skin damage, resulting in sores and cracks that increase the risk of infection. Managing AD is particularly challenging, especially for children, who are the most commonly affected group. Although AD is not curable, various treatments are available to alleviate its symptoms. These treatments typically include topical moisturizers and medications such as corticosteroid creams and antihistamines.^4^ However, the long-term use of these medications, particularly corticosteroids, has raised safety concerns within the medical and scientific communities.^3^

In addition to AD, diabetes mellitus is another chronic disease frequently associated with skin disorders.^5^ Diabetic patients are susceptible to a variety of skin conditions, including acanthosis nigricans, diabetic dermopathy, and allergic reactions, which further compromise their quality of life. Moreover, skin wounds and ulcers in diabetic patients are prone to impaired and delayed healing due to macrophage dysregulation caused by the disease.^6^ This condition reduces mobility, especially when wounds or ulcers affect the lower limbs, and increases susceptibility to infections and other complications.^7^ In severe cases, invasive procedures such as amputations may be required, significantly increasing mortality risks.^8^

To address the challenges of wound healing in diabetic and AD-affected skin, various strategies have been explored, including the use of dressing materials and surgical procedures, often combined with medication. However, many of these approaches have shown limited effectiveness, underscoring the need for new therapies and innovative approaches.^3,9^ Recently, dressing materials from biopolymers such as cellulose, starch, and alginate have emerged as promising alternatives for treating skin disorders associated with diseases like AD and diabetes. Biopolymers offer several advantages, including compatibility with biological systems, biodegradability, low toxicity, weak immunogenicity, and stability across a wide pH range.^10^ Additionally, their hydrophilic nature helps maintain moisture at the treatment site. It prevents tissue maceration by removing excess exudate, which is crucial for enhancing cell proliferation, vascularization, and re-epithelialization at wound sites.^11^ Furthermore, biopolymers’ processability and derivatization potential enable the design of materials with various shapes, morphologies, and properties tailored to specific medical needs.

Among biopolymers, cellulose and its derivatives have been extensively used in creating dressing materials for treating AD and diabetic wounds. For example, Park et al.^12^ developed a pH-responsive hydrogel based on carboxymethyl cellulose that acts as a transdermal release system for delivering naringenin, a flavonoid known to reduce AD symptoms by inhibiting inflammation and enhancing immunity. Similarly, Wang et al.^13^ created a thermoresponsive composite hydrogel using carboxymethyl cellulose, which not only maintains skin moisture but also delivers Cortex Moutan, a traditional Chinese medicine, to treat AD. Cellulose-based hydrogels have also been developed for diabetic wound care. Cheng and collaborators^14^ designed an elastomeric hydrogel composed of hydroxyethyl cellulose, silk nanofibers, magnesium ions, and glycerol, which was shown to accelerate diabetic wound healing. Even raw cellulose has been utilized effectively, as demonstrated by Voss et al.,^15^ who used cellulose fibers from rice husks to create films embedded with propolis and vitamin C, successfully accelerating wound healing in mice while controlling bacterial infections at the wound site.

Nanocellulose, including nanocrystals and nanofibrils, in preparing biomaterials for wound treatment has seen significant growth in recent years.^16^ However, nanocellulose often plays a secondary role, typically being used as a reinforcement agent to improve mechanical properties^17^ or enhance the hydrophilicity of the materials.^18^ Moreover, while exhibiting attractive physicochemical and biological properties, many nanocellulose-based materials remain far from practical application. In this study, we propose using colloidal cellulose nanocrystals (CNC) suspensions as a sprayable coating for treating AD and diabetic wounds, intending to improve treatment outcomes and mitigate the associated symptoms and risks. Unlike conventional methods that produce rigid, nonconformal dressings (such as films and hydrogels) that struggle to cover wounds with irregular shapes, sizes, and depths, sprayable coatings offer a flexible and effective alternative.^19^ Additionally, this approach could reduce the need for frequent dressing changes.

Herein, CNC were extracted from the endosperm of ivory nuts, the seeds of a palm species native to Ecuador.^20^ During the extraction process, mannan (MN), a branched polysaccharide with film- and coating-forming capacity and various bioactive properties,^21^ was also obtained alongside the CNC. We hypothesize that the presence of MN in the CNC suspensions may enhance the performance of the sprayable coatings. To evaluate this hypothesis, we conducted a series of *in vivo* and *ex vivo* evaluations using animal models where 2,4-dinitrochlorobenzene (DNCB) was used to induce AD-like symptoms and streptozotocin (STZ) was used to induce diabetes in mice. To the best of our knowledge, the literature has not reported this direct approach of using a sprayable CNC suspension to treat these skin diseases and wounds.

## 2 Experimental Section

### 2.1 Materials

CNC and MN were obtained from ivory nut flour according to a modified protocol previously described by Carvajal-Barriga et al.^20^ Detailed extraction procedures are provided in Section 2.2. 2,4-Dinitrochlorobenzene (DNCB) and streptozotocin (STZ) were purchased from Sigma-Aldrich (USA). Commercial topical hydrocortisone (HC) cream (1% w/w, Bayer) was purchased from a drugstore (Pelotas, Brazil). Each gram of this cream contains 11.2 mg of HC acetate and excipients (macrogol 400 stearate, stearyl alcohol, liquid petrolatum, white petrolatum, disodium edetate, carbomer 980, sodium hydroxide, methylparaben, propylparaben, and purified water). All other chemicals used in the biochemical assays were of analytical grade and were obtained from standard commercial suppliers.

### 2.2 Extraction of CNC and MN

CNC was extracted from ivory nut endosperm by grinding Tagua seeds to a particle size of <200 μm. The resulting flour (20 g) was subjected to acid hydrolysis in 8 mol/L H_2_SO_4_ at a 1:10 ratio (flour/acid) at 60 °C for 2.5 h under constant stirring (300 rpm). The reaction was stopped by adding an equal volume of deionized water, followed by centrifugation (3000 rpm, 10 min). The supernatant was discarded, and the pellet was resuspended in deionized water and dialyzed for 1 week until the pH reached 5. MN were obtained by alkaline hydrolysis of ivory nut flour using 5% w/v NaOH at room temperature overnight, followed by precipitation with 70% v/v ethanol and successive washing with distilled water until neutral pH. It is important to note that the nanocrystals are formed during the hydrolysis steps: H_2_SO_4_ selectively hydrolyzes the amorphous regions of cellulose, leaving behind the highly ordered crystalline domains that constitute CNC, while alkaline hydrolysis removes amorphous fractions of mannan, yielding ribbon-like MN nanocrystals. The subsequent centrifugation, dialysis, and sonication steps serve only for purification, neutralization, and dispersion, ensuring colloidal stability, but they do not induce nanocrystal formation.

### 2.3 Preparation of CNC and CNC/MN Suspensions

The concentrations of the three colloidal suspensions were adjusted to 0.70% w/v using a stabilizing vehicle solution containing deionized water and 0.25% glycerol (v/v). Table 1 details the composition and characteristics of the colloidal suspensions.

**Table 1:** Sample Coding, Composition, and Characteristics of the Prepared Colloidal Suspensions

sample	CNC (% w/v)	MN (% w/v)	CNC/MN ratio (w/w)	pH	viscosity (cP)
PMM-AC1	0.35	0.35	1:1	3.5	50
PMN-AC1	0.60	0.10	6:1	3.5	20
ENC-01	0.70	-	-	3.5	20

### 2.4 Preclinical Experimental Models

AD model was performed using BALB/c female mice (6–8 weeks old), and the study of diabetic wound healing was conducted using adult male Swiss mice (6–8 weeks old) from a local breeding colony. The animals were exposed to a 12 h light/dark cycle (with light from 7 a.m.) at 22 ± 1 °C and had free access to food and water. Animal experiments were approved for the experimental study obtained from the Ethical Research Commission of the Federal University of Pelotas (CEUA protocol No. 041709/2022–37, and protocol No. 023530/2022–06), according to the National Institutes of Health guidelines for the care and use of laboratory animals (NIH Publications No. 8023, revised 1978). The minimum number of animals required to demonstrate the consistent effects of the drug treatment regimen was used, and all efforts were made to ensure their comfort.

### 2.5 AD Model: In Vivo and Ex Vivo Assays

The preclinical AD model was performed according to the previous methodology described,^22^ using DNCB to induce AD-like skin lesions. Initially, the mice were divided into six experimental groups (seven per group).

(I) **Control group:** Mice’s ears and dorsal region received the solution without DNCB, and no treatment;
(II) **DNCB group:** Mice’s ears and dorsal region were sensitized with DNCB solution, and received the cutaneous application of vehicle of the CNC and MN (deionized water and 0.25% glycerol (v/v));
(III) **TTO1 group:** Mice’s ears and dorsal region were sensitized with DNCB solution, and received the cutaneous application of TTO1 (PMM-AC1);
(IV) **TTO2 group:** Mice’s ears and dorsal region were sensitized with DNCB solution, and received the cutaneous application of TTO2 (PMN-AC1);
(V) **TTO3 group:** Mice’s ears and dorsal region were sensitized with DNCB solution, and received the cutaneous application of TTO3 (ENC-01);
(VI) **HC group:** Mice’s ears and dorsal region were sensitized with DNCB solution, and received the cutaneous application of hydrocortisone (HC).

Figure 1 illustrates a schematic design of the experimental protocol. In the sensitization phase, the dorsal region of each animal was shaved, and 200 μL of 0.5% (v/v) DNCB in acetone/olive oil (ratio: 3:1, v/v) was applied on days 1–3. Subsequently, on days 14, 17, 20, 23, 26, and 29, the animals were sensitized again with 200 μL of 1% (v/v) DNCB in the dorsal region and 20 μL on the right ear.

**Figure 1:**
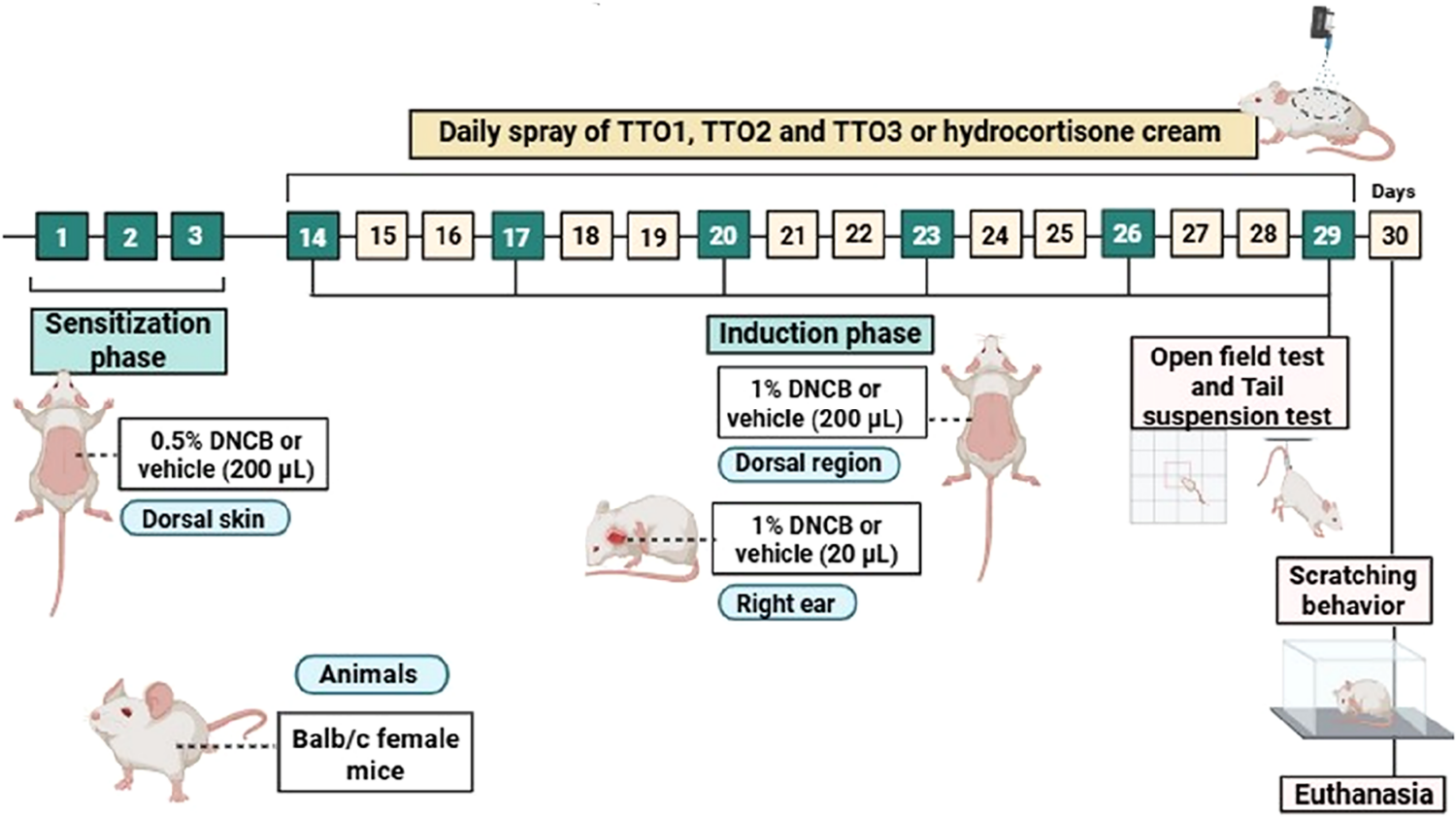
Summary of the experimental protocol for developing AD-like skin lesions in DNCB-sensitized mice.

The spray application of treatments (TTO1, TTO2, and TTO3) or vehicle solution followed a standardized procedure, where approximately 60 μL of each colloidal suspension was uniformly sprayed onto the injured site once a day from a distance of 15 cm from day 14 to day 29. Regarding the reference drug application (HC), 0.5 g of cream was applied only once a day to the injured site. On days 29 and 30, behavioral tests were conducted before euthanasia, followed by the collection of back and ear samples for further analysis.

#### 2.5.1 Assessment of the Emotional Domain

On the 29th day of the experimental protocol, the tail suspension test was conducted in a controlled, quiet environment, with the total duration of immobility serving as the primary parameter to assess “behavioral despair” in rodents.^23^ For this, each mouse was suspended by its tail using adhesive tape and positioned 50 cm above the floor. Over 6 min, immobility time (in seconds) was recorded. Mice were considered immobile only when they hung passively and remained completely motionless. The increase in the duration of immobility is indicative of a depressive phenotype.

#### 2.5.2 Assessment of Locomotor and Exploratory Performance

The open field test evaluated general locomotor activity and exploratory behavior, excluding nonspecific effects such as psychostimulatory activity.^24^ The apparatus, constructed of plywood (30 cm height × 45 cm depth × 45 cm width), had its floor divided into nine equal quadrants (3 rows of 3) using adhesive tape markers. Each animal was placed at the center of the apparatus and observed for 4 min. Locomotor activity (measured as the number of segments crossed with all four paws) and exploratory behavior (measured as the number of rearing on the hind limbs) were recorded on the 29th day of the experimental protocol. The apparatus was cleaned with 30% ethanol after each session.

#### 2.5.3 Scratching Behavior

Pruritus caused by DNCB-induced AD-like lesions in mice was assessed on day 30 of the protocol, following the previous methodology described.^25^ During a 20 min observation period, the time spent by mice rubbing their nose, ears, and dorsal skin with their hind paws was recorded to quantify scratching behavior.

#### 2.5.4 Clinical Skin Severity Scores

The evaluation of lesions in dorsal skin samples and the assignment of severity scores were performed by analyzing photographs taken on the last day of the experiment. Five signs of skin lesions were assessed: pruritus/itching, erythema/hemorrhage, edema, excoriation/erosion, and scaling/dryness. Each symptom was classified on a scale of 0 (absent), 1 (mild), 2 (moderate), and 3 (severe) according to the previously described method.^25^

#### 2.5.5 Evaluation of Inflammatory Parameters

##### 2.5.5.1 Spleen Index and Ear Swelling

The spleen weight and length alterations were assessed using an analytical balance (AUY-SHIMADZU, Japan) and a digital caliper. Furthermore, both ears were sectioned at the base, and the degree of swelling between the control ear (left) and the DNCB-sensitized ear (right) was quantified using the analytical balance.

##### 2.5.5.2 Determination of Myeloperoxidase (MPO) Activity

The evaluation of MPO activity was conducted based in the previously described method with minor modifications.^26^ Tissues from the back and ears were extracted and homogenized in phosphate-buffered saline (PBS) (20 mmol/L, pH 7.4) containing 0.1 mmol/L ethylenediaminetetraacetic acid (EDTA). The homogenates were then centrifuged at 900 g for 10 min at 4 °C, with the pellet being discarded. The supernatant (S_1_ fraction) underwent a second centrifugation at 24,000*g* for 15 min at 4 °C, resulting in the final pellet (P_2_), which was resuspended in 50 mmol/L potassium phosphate buffer (pH 6.0) containing 0.5% (w/v) hexadecyltrimethylammonium bromide. Following three freeze–thaw cycles, MPO activity was determined by adding 100 μL of the P_2_ suspension to a reaction medium containing the buffer and 1.5 mmol/L *N*,*N*,*N*′,*N*′-tetramethylbenzidine. The reaction was initiated by adding 0.01% (v/v) hydrogen peroxide (H_2_O_2_), and absorbance was measured at 655 nm at 37 °C. Results were expressed as optical density (OD) per mg of protein per minute. Protein concentration was quantified using the Bradford method, with bovine serum albumin (1 g/L) as the standard.^27^

##### 2.5.5.3 Histological Analysis

Histological analysis assessed morphological changes in the tissue and cell migration to the affected back areas.^28^ Dorsal skin fragments were collected and fixed in a 10% buffered formalin solution. The samples were embedded in paraffin, sectioned into 3–4 μm slices, stained with hematoxylin and eosin (H.E.), and examined under a light microscope. Comparisons between the treatment and control groups were based on intraepidermal pustules, crust formation, inflammatory infiltrate in both the superficial and deep dermis, and subcutaneous inflammatory infiltrate. The severity of these findings was evaluated qualitatively and classified as discrete (low), moderate (medium), or severe (high). Two pathologists independently examined the slides, and the final classification was determined collaboratively.

#### 2.5.6 Oxidative Parameters

Dorsal skin samples and the right ear were homogenized in a 50 mM Tris-HCl buffer (pH 7.4, 1:10, w/v) to assess oxidative stress parameters. The homogenate was centrifuged at 900*g* for 10 min, and the resulting supernatant (S_1_) was collected. Aliquots of S_1_ were then used to measure levels of reactive species (RS), thiobarbituric acid-reactive substances (TBARS), nonprotein thiol groups (NPSH), and catalase (CAT) enzyme activity.

##### 2.5.6.1 RS Levels

The levels of RS were measured using a spectrofluorimetric method previously described.^29,30^ An aliquot of S_1_ was incubated with 1 mM 2′,7′-dichlorofluorescein diacetate (DCFH-DA) and 10 mM Tris HCl (pH 7.4) for 60 min. The intracellular RS concentration was determined by oxidizing DCFH-DA to produce fluorescent dichlorofluorescein (DCF). The fluorescence intensity of DCF was measured at an emission wavelength of 525 nm, with excitation at 488 nm, using a Shimadzu RF-5301 PC fluorometer. Results were expressed as arbitrary fluorescence units (UF).

##### 2.5.6.2 TBARS Levels

The levels of TBARS, used as a marker for lipid peroxidation, were quantified following the methodology previously described.^31^ In this procedure, an aliquot of S_1_ was combined with a solution containing 0.8% thiobarbituric acid (pH 3.4), an acetic acid buffer, and 8.1% sodium dodecyl sulfate. The reaction mixture was then incubated for 2 h at 95 °C, after which the absorbance was measured at 532 nm. The results were expressed as nmol of malondialdehyde (MDA)/ mg of protein.

##### 2.5.6.3 NPSH Levels

Nonprotein thiol (NPSH) levels were determined using Ellman’s method.^32^ A 1:2 volume ratio of the S_1_ sample was combined with 10% trichloroacetic acid. Following centrifugation at 3000 rpm for 10 min, the protein pellet was discarded, and the free thiol groups were measured in the resulting clear supernatant. An aliquot of this supernatant was then mixed with 1 M potassium phosphate buffer (pH 7.4) and 10 mM 5,5′-dithio-bis (2-nitrobenzoic acid). The color reaction was analyzed at 412 nm. NPSH levels were reported as nmol NPSH/g of tissue.

##### 2.5.6.4 CAT Activity

CAT activity was determined using the spectrophotometric method by Aebi.^33^ This method tracks the rate of H_2_O_2_ decomposition in the S_1_ aliquot at 240 nm. The reaction was initiated by adding H_2_O_2_ and S_1_ to a system containing a 50 mM potassium phosphate buffer (pH 7.0). The results were reported as CAT units per milligram of protein (U CAT/mg protein), where 1 unit corresponds to the decomposition of 1 μmol of H_2_O_2_ per minute at pH 7 and 25 °C.

### 2.6 Diabetic Wound Healing: In Vivo and Ex Vivo Assays

As described below, the mice were separated into five groups (7 animals per group).

(I) **Control group:** No diabetic wound-induced and received no treatment;
(II) **STZ group:** Diabetic wound-induced, and received the cutaneous application of the vehicle of the CNC and MN (deionized water and 0.25% glycerol (v/v));
(III) **TTO1 group:** Diabetic wound-induced and received the cutaneous application of TTO1 (PMM-AC1);
(IV) **TTO2 group:** Diabetic wound-induced and received the cutaneous application of TTO2 (PMN-AC1);
(V) **TTO3 group:** Diabetic wound-induced and received the cutaneous application of TTO3 (ENC-01).

Figure 2 shows a schematic design of the experimental protocol. The diabetic phenotype was induced in the mice as described in preceding study.^34^ In summary, animals received daily intraperitoneal (i.p.) injections of STZ (100 mg/kg) for three consecutive days, with STZ freshly prepared in a citrate buffer (0.1 M, pH 4.4). Control animals received citrate buffer injections (10 mL/kg, i.p.) for 3 days. One week after STZ administration, fasting blood glucose levels were measured (day 0) using an Accu-Chek Advantage glucose monitor (Roche Diagnostic Corporation, IN), with values above 200 mg/dL indicating diabetes.^15^

**Figure 2:**
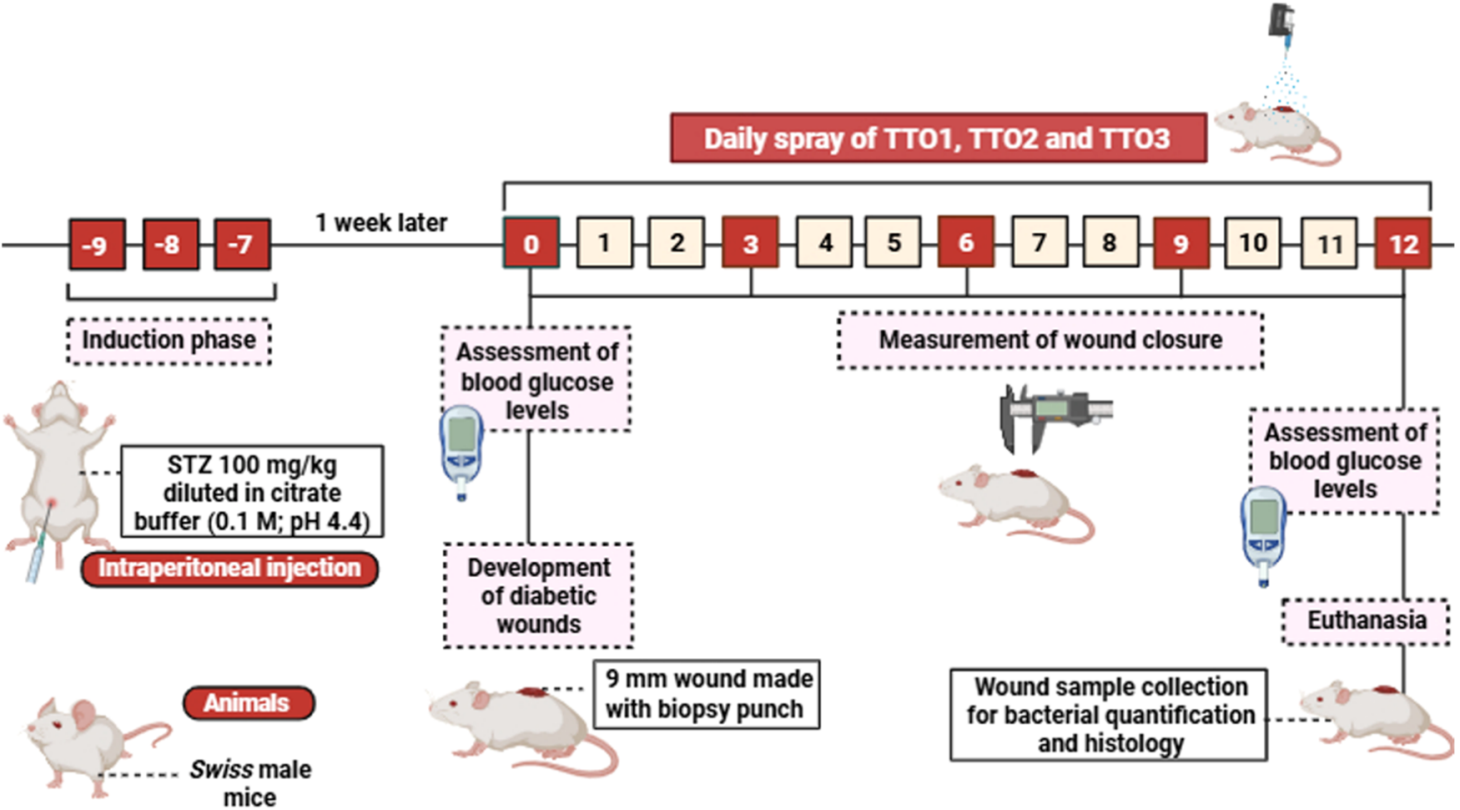
Summary of the experimental protocol for the induction of diabetic wounds in mice.

After confirming diabetic induction, mice were anesthetized with isoflurane, and their dorsal skin was shaved. A 9 mm wound was created in each mouse’s back using a biopsy punch. Regarding the treatments, a spray application was used, with approximately 60 μL of each colloidal suspension uniformly applied to the wound area from a distance of 15 cm. Treatments were administered until the 12th day of the experimental protocol. Wound closure was assessed on days 3, 6, 9, and 12, expressed as a percentage of wound area closure. On the 12th day, blood glucose levels were measured, and animals were euthanized via isoflurane inhalation. Wound samples were collected for bacterial quantification and histopathological evaluation.

#### 2.6.1 Quantification of Bacteria in Treated Wounds

The swab was pressed against the tube wall containing 1 mL of sterile saline multiple times for 30 s to ensure the transfer of bacteria from the swab to the solution. Then, 100 μL of this solution was used in serial 10-fold dilutions in sterile saline. Immediately, 1 μL of each dilution was added to a sterile Petri dish with agar Brain-Heart Infusion with a calibrated loop, which was incubated for 24 h at 37 °C. Furthermore, a sterile saline solution was inoculated into Petri dishes and used as the control. After incubation, microbial colonies were counted and converted to log colony-forming units (CFU)/mL.

#### 2.6.2 Histological Analysis

Wound samples for histological analysis were taken after the euthanasia of the animals on day 12 of the experimental protocol. The tissue samples were immediately collected and fixed in 10% buffered formalin. For evaluation under optical microscopy, the tissues were embedded in paraffin, sectioned into 3–4 μm slices, and stained with H.E. and Masson’s Trichrome (M.T.) to enhance collagen visualization. The inflammatory response was classified as mild, moderate, or severe. Collagen production, assessed using M.T. staining, was categorized as weak, moderate, or high.^35^ Two pathologists independently examined the slides, and the final classification was determined collaboratively.

### 2.7 Physicochemical Characterization of Colloidal Suspensions

The characterization methods are detailed in the Supporting Information file.

### 2.8 Statistical Analysis

All experimental results are presented as the mean ± standard error of the mean (S.E.M.). The statistical analyses were conducted using GraphPad Prism Software version 8.0 (San Diego, CA, USA). A Gaussian distribution was evaluated using the Shapiro-Wilk normality test. Data were analyzed by one-way analysis of variance (ANOVA), followed by a Tukey post hoc test when appropriate. Probability values less than 0.05 (*p* < 0.05) were considered statistically significant.

## 3 Results and Discussion

### 3.1 Evaluations of the Therapeutic Effects of TTO1, TTO2, TTO3, and HC in the Atopic Dermatitis

#### 3.1.1 Effect of TTO1, TTO2, TTO3, and HC in the Clinical Signs of AD-like Skin Lesions in Mice

DNCB is a chlorinated aromatic compound classified as a hapten and is widely employed as a model for contact sensitization in mice.^36^ Repeated exposure to DNCB elicits a cutaneous inflammatory response mediated by the adaptive immune system, leading to skin lesions resembling those seen in AD.^37^ Furthermore, this induction alters the physicochemical properties of the epidermis, contributing to dryness, thickening, swelling, edema, and erythema. In certain cases, this heightened response may culminate in anaphylactic reactions.^38^

Figure 3 presents the effects of DNCB sensitization and the cutaneous application of TTO1, TTO2, TTO3, and HC cream on the severity of skin lesions (Figure 3A,B) and scratching behavior (Figure 3C,D) in mice. A one-way analysis of variance (ANOVA), followed by Tukey’s post hoc test, revealed that DNCB exposure significantly increased skin severity scores compared with the control group (*F*_(5,36)_ = 21.37, *p* < 0.0001). These animals presented skin changes, including dryness, hemorrhage, excoriation, and edema. Moreover, DNCB-sensitized animals exhibited a short latency to the first itching episode (*F*_(5,36)_ = 13.01, *p* < 0.0001) and a longer total itching time (*F*_(5,36)_ = 44.28, *p* < 0.0001) relative to controls, reflecting hallmark symptoms of AD-like. These findings demonstrate that repeated exposure to DNCB successfully mimicked phenotypic features of AD in mice, including allergic responses analogous to those observed in patients with this condition.

**Figure 3:**
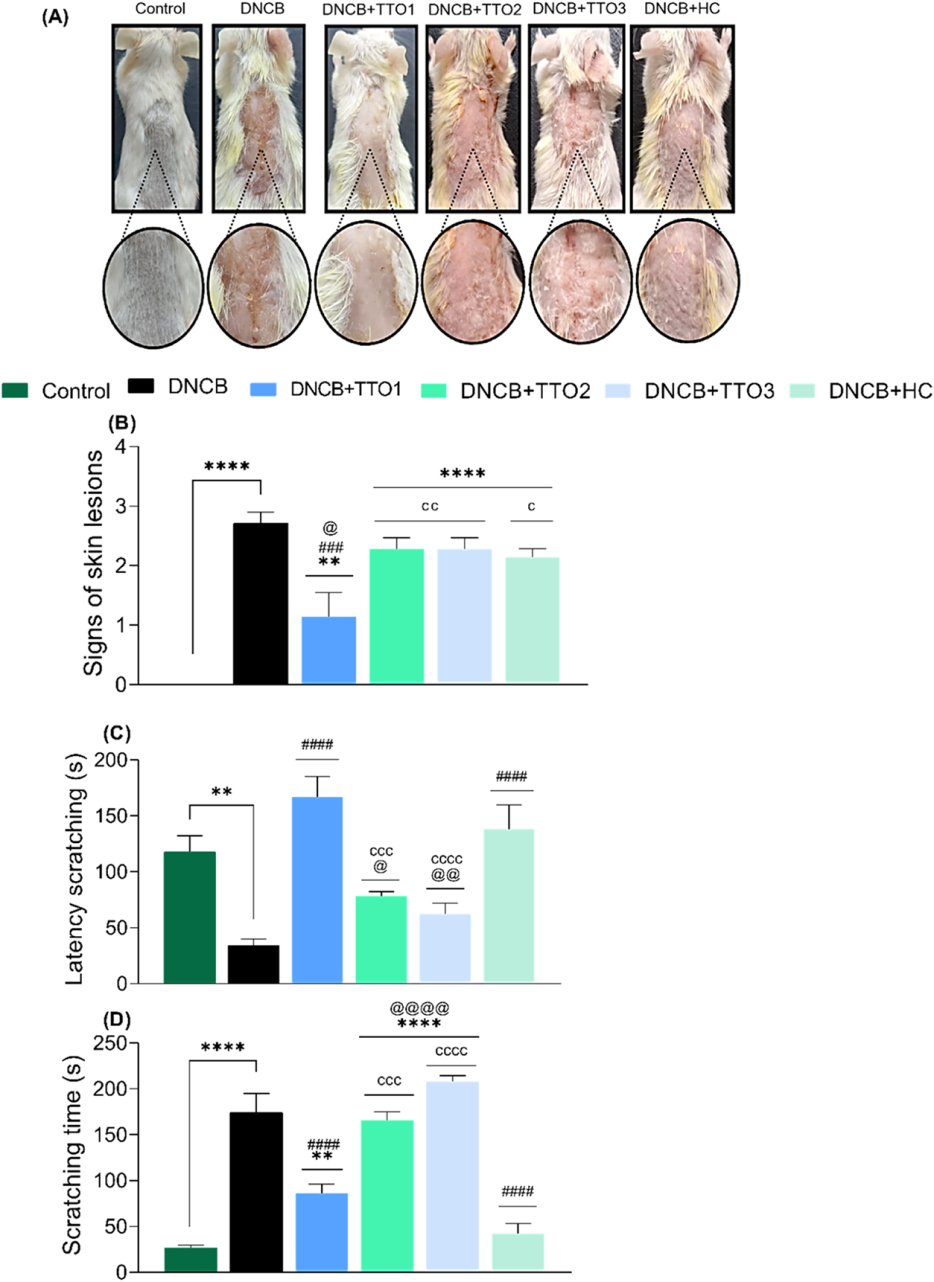
Effects of cutaneous application of TTO1, TTO2, TTO3, and HC in the atopic dermatitis-like (AD-like) symptoms in mice induced by DNCB. Representative photographic images of dorsal skin (A), skin lesion scores (B), latency to the first episode of itching (**C**), and scratching time (D) after DNCB-sensitization. Data means ± SEM of 7 animals per group (one-way ANOVA followed by Tukey’s test). (**) *p* < 0.01, and (****) *p* < 0.0001 compared with the control group; (###) *p* < 0.001, and (####) *p* < 0.0001 compared with the DNCB group, and (@) *p* < 0.05, (@@) *p* < 0.01, and (@@@@) *p* < 0.0001 compared with the hydrocortisone (HC) group, and (c) *p* < 0.05, (cc) *p* < 0.01 compared with the TTO1 group.

Daily cutaneous administration of TTO1 mitigated the severity of skin lesions, prolonged the latency to the onset of itching episodes, and reduced the total time spent itching induced by DNCB. In addition, the TTO1 significantly improved clinical and behavioral parameters compared to those detected with HC cream. The results presented here corroborate previous studies that demonstrate the potential of CNC-MN in the controlled release of drugs, attributed to its physicochemical properties that favor therapeutic efficacy.^39,40^ The TTO2 and TTO3 were not able to attenuate the evaluated parameters.

#### 3.1.2 Effect of TTO1, TTO2, TTO3, and HC n in the Neuropsychiatric Disorders Associated with AD-like in Mice

Like other dermatological conditions, AD adversely affects patients’ quality of life, contributing to low self-esteem, social isolation, and psychological stress.^41^ Studies have consistently reported a higher prevalence of depression in both adults and children with AD, highlighting a potential link between chronic inflammation and emotional disorders. Notably, symptoms such as itching, insomnia, and excoriation are directly associated with the onset or worsening of depressive states.^42^ In the present study, sensitization with DNCB significantly increased the total immobility time in the tail suspension test (Figure 4A) (*F*_(5,36)_ = 7.873, *p* < 0.0001), compared with the control group, indicating that the induction elicited a depressive-like phenotype in the animals. These findings align with a previous study demonstrating the ability of DNCB to induce depressive-like behavior in rodent models.^43^

**Figure 4:**
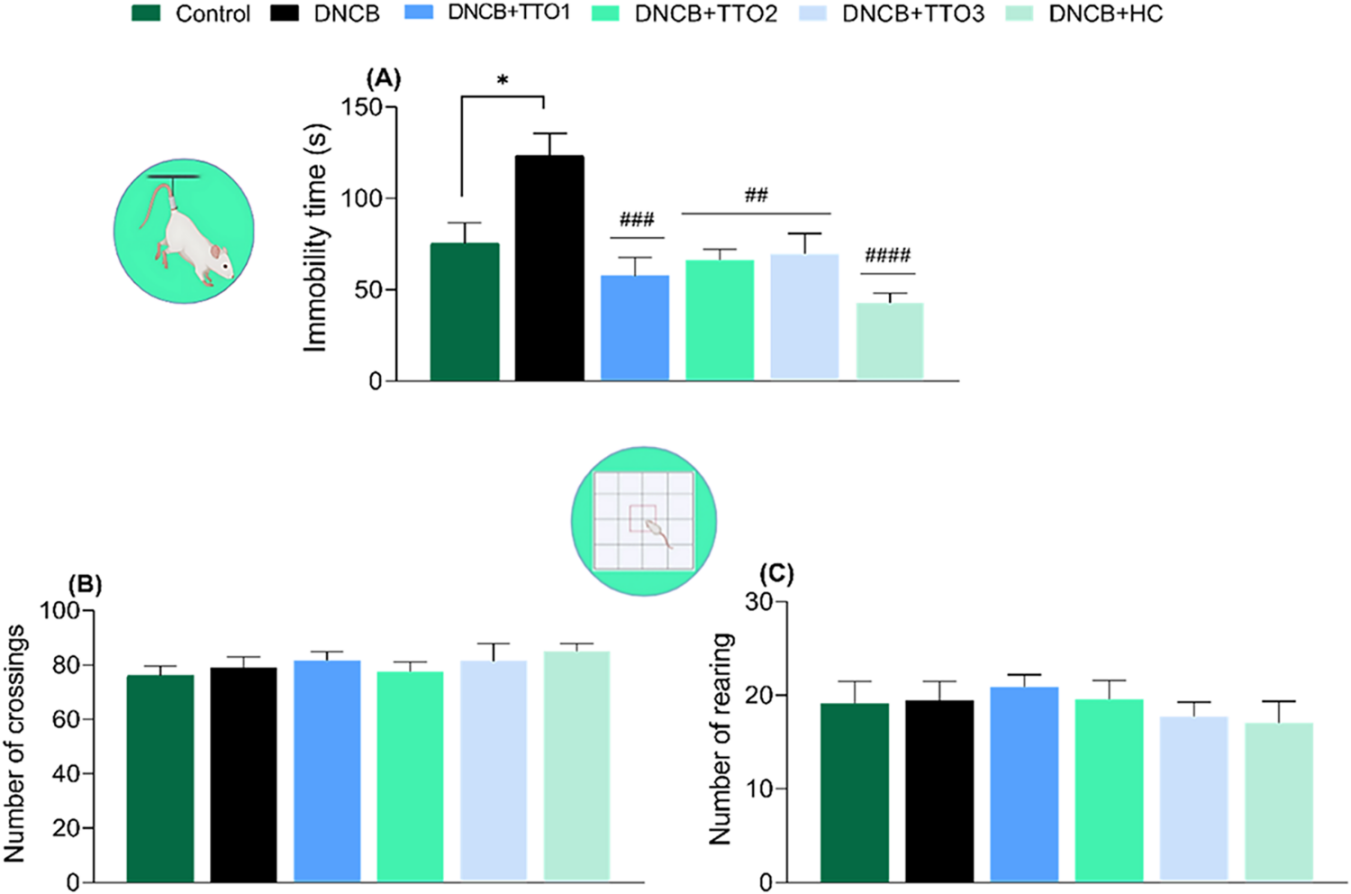
Effects of cutaneous administration of TTO1, TTO2, TTO3, and HC in the tail suspension and open field tests in DNCB-induced mice. Represents the immobility time (A), the number of crossings (B), and the number of rearing (C). Data means ± SEM of 7 animals per group (one-way ANOVA followed by Tukey’s test). (*) *p* < 0.05 compared with the control group. (##) *p* < 0.01 and (###) *p* < 0.001 compared with the DNCB group.

In this context, daily cutaneous administration of TTO1, TTO2, TTO3, and HC reduced the immobility time of mice compared with the DNCB group. This antidepressant-like effect is likely related to the ability of these treatments to alleviate the main symptoms associated with AD. Previous evidence has demonstrated that treatments effective in reducing AD symptoms may also attenuate depressive-like phenotypes in animal models.^43,44^ Furthermore, it is important to highlight that neither DNCB-sensitized nor the cutaneous administration of TTO1, TTO2, TTO3, and HC altered the animals’ locomotor (Figure 4B) (*F*_(5,36)_ = 0.6071, *p* > 0. 05) or exploratory activities (Figure 4C) (*F*_(5,36)_ = 0.4954, *p* > 0.05) in the open field test. This finding suggests that, while exerting their antidepressant-like effects, the treatments did not induce sedation, a common adverse effect associated with some clinically used antidepressants.^45^

#### 3.1.3 Effect of Cutaneous Administration of TTO1, TTO2, TTO3, and HC in the Inflammatory Responses of AD-like Skin Lesions in Mice

The pathophysiology of AD is linked to multiple systemic factors, most of which involve extensive immunological dysregulations. Evidence indicates that abnormalities begin in the innate immune system, the body’s first line of defense, responsible for rapid and nonspecific protection against external agents. The spleen, the largest secondary lymphoid organ in the human body, acts as a host for various immunological functions.^46^ Among the central roles played by this organ are the filtration and recycling of blood cells and initiating the adaptive immune response, with the production of immune cells.^47^ In this study, we measured the mass and length of the spleen of mice on day 30 to assess splenomegaly, an indicator of immunological abnormalities.

Figure 5 shows that exposure to DNCB causes a significant increase in the mass (Figure 6A) (*F*_(5,36)_ = 98.06, *p* < 0.0001) and length of the spleen of mice (Figure 5B) (*F*_(5,37)_ = 15.45, *p* < 0.0001) compared with the control group. In contrast, cutaneous application of TTO1 and TTO2, as well as HC, decreased the changes in spleen mass (Figure 5A). However, only HC decreased the changes in spleen length in mice compared with the DNCB group (Figure 5B).

**Figure 5:**
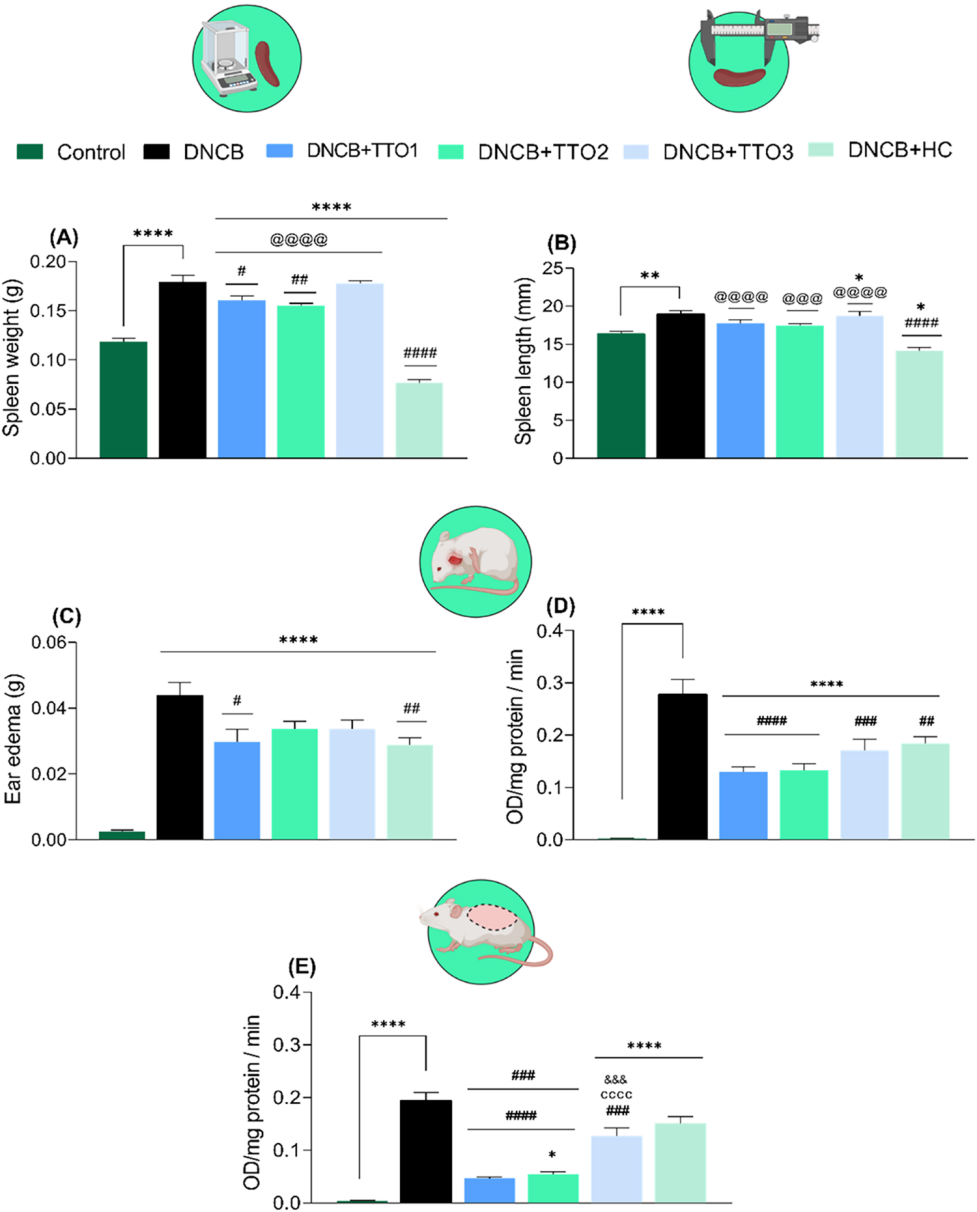
Effects of cutaneous administration of TTO1, TTO2, TTO3, and HC in the atopic-dermatitis-like (AD-like) symptoms in mice induced by DNCB-sensitization. Spleen weight (A), spleen length (B), ear swelling (C), ear MPO activity (D), and dorsal skin MPO activity (E). Data means ± SEM of 7 animals per group (one-way ANOVA followed by Tukey’s test). (*) *p* < 0.05, (**) *p* < 0.01 and (****) *p* < 0.0001 compared with the control group; (#) *p* < 0.05, (##) *p* < 0.01, (###) *p* < 0.001 and (####) *p* < 0.0001 compared with the DNCB group; (@@@) *p* < 0.001 and (@@@@) *p* < 0.0001 compared with the hydrocortisone (HC) group; (cccc) compared with the DNCB + TTO1 group; (&&&&) compared with the DNCB + TTO2 group.

**Figure 6:**
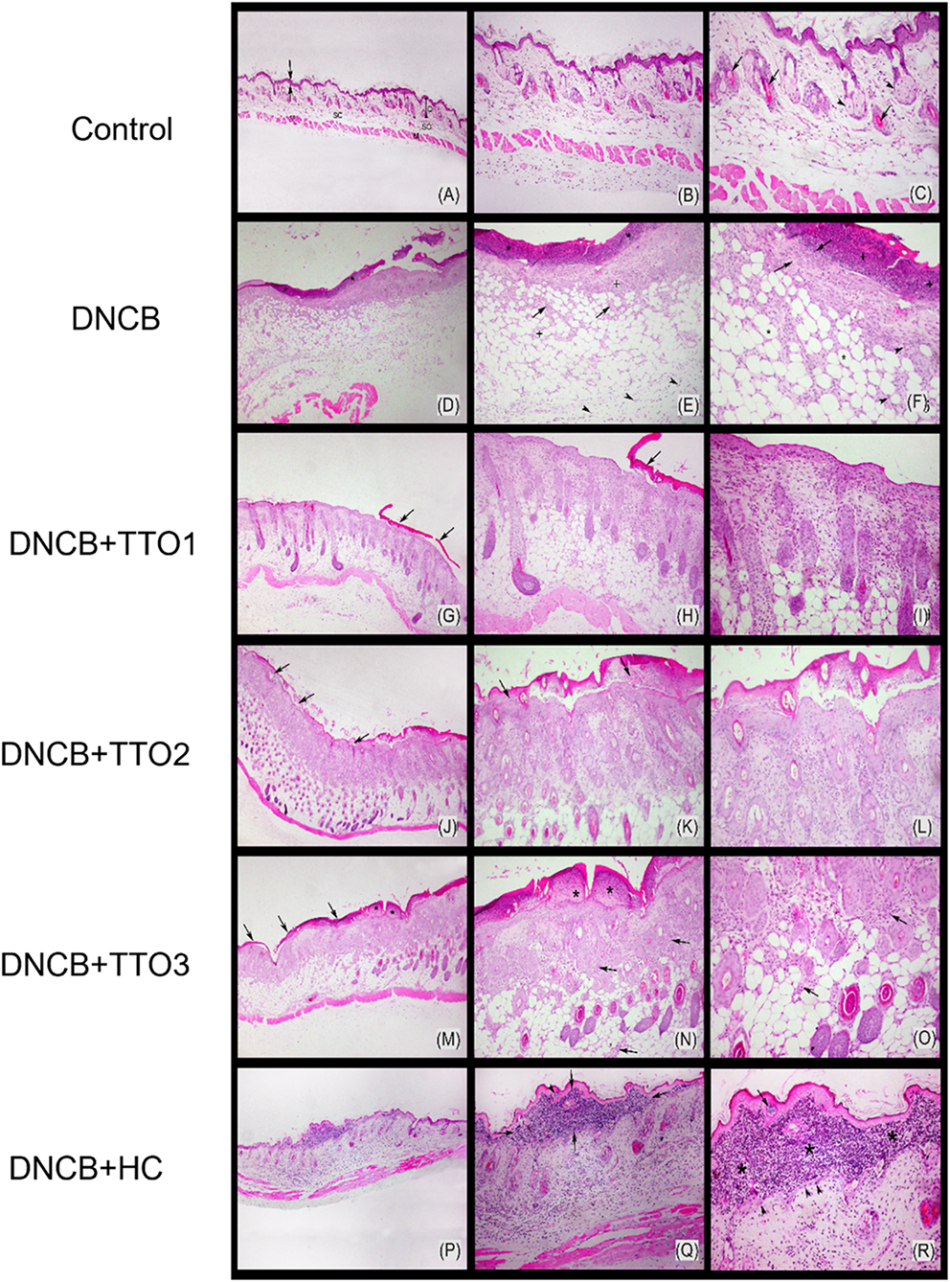
Histological profiles of the dorsal skin of mice after DNCB-sensitization and cutaneous administration of TTO1, TTO2, TTO3, and HC using HE staining. Control mouse–unimpaired skin, showing the epidermis (between arrows), dermis (D), subcutaneous tissue (SC), and muscular layer (M), as well as skin appendages such as sebaceous glands (arrowhead) and hair follicle (arrow), HE. 40× objective lens (A), 100× objective lens (B), and 200× objective lens (C). Mouse sensitized with DNCB—Skin shows crust formation (*) and abundant inflammatory infiltrate of neutrophils (arrow) and mononuclear cells (arrowhead) extending from the epidermis to the subcutaneous layer, in addition to many dilated lymphatic vessels (+), HE. 40× objective lens (D), 100× objective lens (E), and 200× objective lens (F). DNCB-sensitized and received cutaneous administration of TTO1—Skin with mild parakeratotic hyperkeratosis, crust (arrow) presence, and mild inflammatory infiltration of neutrophils and some mononuclear cells, HE. 40× objective lens. (G), 100× objective lens (H), and 200× objective lens (I). DNCB-sensitized and received cutaneous administration of TTO2—Skin showing intraepidermal pustules (arrow), moderate parakeratotic hyperkeratosis, and moderate inflammatory infiltrate of neutrophils and mononuclear cells in the superficial dermis, HE. 40× objective lens. (J), 100× objective lens (K), and 200× objective lens (L). DNCB-sensitized and received the cutaneous administration of TTO3—Skin with moderate parakeratotic hyperkeratosis, crust formation (arrow), and large intraepidermal pustules (*). Inflammatory infiltrate predominantly of neutrophils in the subcutaneous tissue and in the superficial and deep dermis, HE. 40× objective lens. (M), 100× objective lens (N), and 200× objective lens (O). DNCB-sensitized and received the cutaneous administration of HC. Skin presents an abundant inflammatory infiltrate of neutrophils in the superficial, deep dermis, and subcutaneous tissue. Skin presenting a large intraepidermal pustule (between arrows) and the presence of a bacterial colony (arrowhead). Abundant inflammatory infiltrate of neutrophils and mononuclear cells in the dermis and subcutaneous tissue. Large intraepidermal pustule (*), basal cell layer of the epidermis (arrowhead), and presence of a bacterial colony (arrow), HE. 40× objective lens. (P), 100× objective lens (Q), and 200× objective lens (R).

In the long term, exacerbated cutaneous inflammation can trigger lesions resulting from the inflammatory process. In mice, repeated application of DNCB to the ear induces inflammation characterized by edema and cellular infiltration, primarily of T lymphocytes and macrophages.^48^ The severity of inflammation was measured by ear swelling, and the effects of the treatments are shown in Figure 5C. One-way ANOVA followed by Tukey’s posthoc test revealed that DNCB increased ear edema compared with the control group (Figure 5C) (*F*_(5,36)_ = 24.21, *p* < 0.0001), consistent with DNCB inducing an AD-like phenotype in mice. Cutaneous administrations of TTO1 and HC attenuated ear edema induced by DNCB exposure. On the other hand, TTO2 and TTO3 did not attenuate the parameters evaluated.

The ear edema observed in DNCB-exposed mice reflects one of the main signs of local inflammation in the AD model. MPO enzyme activity was evaluated as a marker of neutrophil infiltration in the inflamed region to better understand the cellular mechanisms underlying this response.^49^ In our study, we showed that DNCB induction caused an increase in MPO activity in the right ear (Figure 5D) (*F*_(5,39)_ = 28.95, *p* < 0.0001) and dorsal skin (Figure 5E) (*F*_(5,36)_ = 48.34, *p* < 0.0001) when compared to the control group. Daily administration of TTO1, TTO2, and TTO3 mitigated the DNCB-induced increase in MPO activity in the dorsal skin (Figure 5E) and right ear (Figure 5D) of the mice. On the other hand, HC attenuated the increase in MPO activity only in the right ear (Figure 5D). The cutaneous application of TTO1 and TTO2 demonstrated superior efficacy in reducing MPO activity in the dorsal skin of the mice, as compared with the HC (positive control). Furthermore, cutaneous application of TTO1 and TTO2 exhibited enhanced performance in reducing MPO activity in the dorsal skin of DNCB-sensitized mice compared to TTO3.

The severity of the morphological alterations observed in the dorsal skin of mice following exposure to DNCB and the respective treatments are show in Figure 6. The dorsal skin of control mice exhibited standard histological architecture (Figure 6A,C). In contrast, the mice sensitized with DNCB displayed nonulcerated skin associated with the formation of subepidermal and intraepidermal pustules within a hyperplastic epidermis (Figure 6F), accompanied by mild parakeratotic hyperkeratosis. The dermis appeared thickened and edematous, with an abundant inflammatory infiltrate of neutrophils in the superficial dermis. In the deeper dermis, vascular congestion and dilated lymphatic vessels were observed. The superficial and deep dermis also exhibited a predominantly neutrophilic inflammatory infiltrate (Figure 6E), with rare macrophages and lymphocytes and crust formation in some areas (Figure 6D).

Cutaneous application of TTO1 demonstrated the best therapeutic response at the end of the experiment. Their skin showed mild parakeratotic hyperkeratosis (Figure 6G), with only a few foci of crust formation (Figure 6H) and a slight neutrophilic inflammatory infiltrate, predominantly restricted to the superficial dermis (Figure 6I). In mice receiving TTO2, histological analysis revealed the presence of intraepidermal pustules (Figure 6J), moderate parakeratotic hyperkeratosis (Figure 6K), and a mild inflammatory response in the superficial dermis (Figure 6L), accompanied by a moderate inflammatory infiltrate composed of neutrophils and mononuclear cells. Conversely, the mice subjected to TTO3 had an inferior response compared to TTO1 and TTO2. These animals exhibited moderate parakeratotic hyperkeratosis, crust formation (Figure 6M), and large intraepidermal pustules (Figure 6N), associated with a predominantly neutrophilic inflammatory infiltrate extending into the dermis and subcutaneous tissue (Figure 6O). These results can be attributed to the presence of MN in the colloidal suspensions used in TTO1 and TTO2. As reported in the literature, MN derived from different sources can exhibit anti-inflammatory properties.^50^

Finally, mice treated with HC cream showed dorsal skin with an abundant neutrophilic inflammatory infiltrate affecting the superficial and deep dermis and subcutaneous tissue (Figure 6P). Large intraepidermal pustules (Figure 6Q) and bacterial colonies (Figure 6R) were also observed.

As observed, the results presented in this study demonstrate that repeated cutaneous sensitization with DNCB induces a significant inflammatory response in the dorsal skin of mice characterized by morphological alterations such as epidermal thickening, formation of intraepidermal pustules, and predominantly neutrophilic inflammatory infiltrate. The use of HC, despite being a widely used treatment for cutaneous inflammatory conditions, showed limited results and was associated with adverse effects, such as the presence of a significant neutrophil infiltrate and formation of intraepidermal pustules, reinforcing the risks of continuous corticosteroid use.^51^

On the other hand, TTO1, TTO2, and TTO3 demonstrated superior therapeutic potential, where TTO1 showed the best response, characterized by a significant reduction in inflammation and skin alterations. These findings may be associated with the intrinsic anti-inflammatory and bioactive properties of the components used in the formulations. Previous studies have shown that nanocellulose-based materials can modulate inflammatory responses, reducing cytokine release and promoting tissue regeneration.^52,53^ Similarly, mannan and its oligosaccharide derivatives exhibit recognized anti-inflammatory effects by attenuating the expression of proinflammatory cytokines such as TNF-α and IL-1β while enhancing IL-10 levels.^54^ Therefore, the combined presence of CNC and MN in the formulations may have contributed synergistically to the observed improvement in the inflammatory profile and the recovery of skin integrity. Overall, these results highlight the potential of the tested treatments as promising corticosteroid alternatives with a lower likelihood of adverse effects. Hence, the present findings contribute to the development of innovative therapeutic strategies for managing cutaneous inflammatory disorders and provide a foundation for future studies aimed at optimizing and validating these formulations.

#### 3.1.4 Effect of Cutaneous Administration of TTO1, TTO2, TTO3, and HC on the Oxidative Status of AD-like Skin Lesions in Mice

The exacerbated chronic inflammation in AD is one of the main factors destabilizing redox homeostasis, leading to an imbalance between RS production and the organism’s antioxidant capacity to neutralize them.^55^ In this study, as shown in Figure 7, animals sensitized with DNCB presented significantly higher levels of RS (Figure 7A) and TBARS (Figure 7B) in the dorsal skin compared with the control group (RS: *F*_(5,36)_ = 59.64, *p* < 0.0001; TBARS: *F*_(5,36)_ = 33.51, *p* < 0.0001). The overproduction of RS reflects the persistent activation of inflammatory processes, marked by the release of pro-inflammatory cytokines and the increase in the activity of peroxidative enzymes, such as MPO. This pro-oxidant environment oxidizes essential biomolecules, including proteins and lipids of the extracellular matrix. Consequently, the products of these oxidative reactions amplify the inflammatory response by activating the expression of genes that encode pro-inflammatory cytokines, generating a continuous cycle that perpetuates the inflammatory process. This mechanism also compromises the integrity of the dermal matrix, leading to skin thickening and loss of elasticity, typical features of AD observed in animals induced with DNCB.

**Figure 7:**
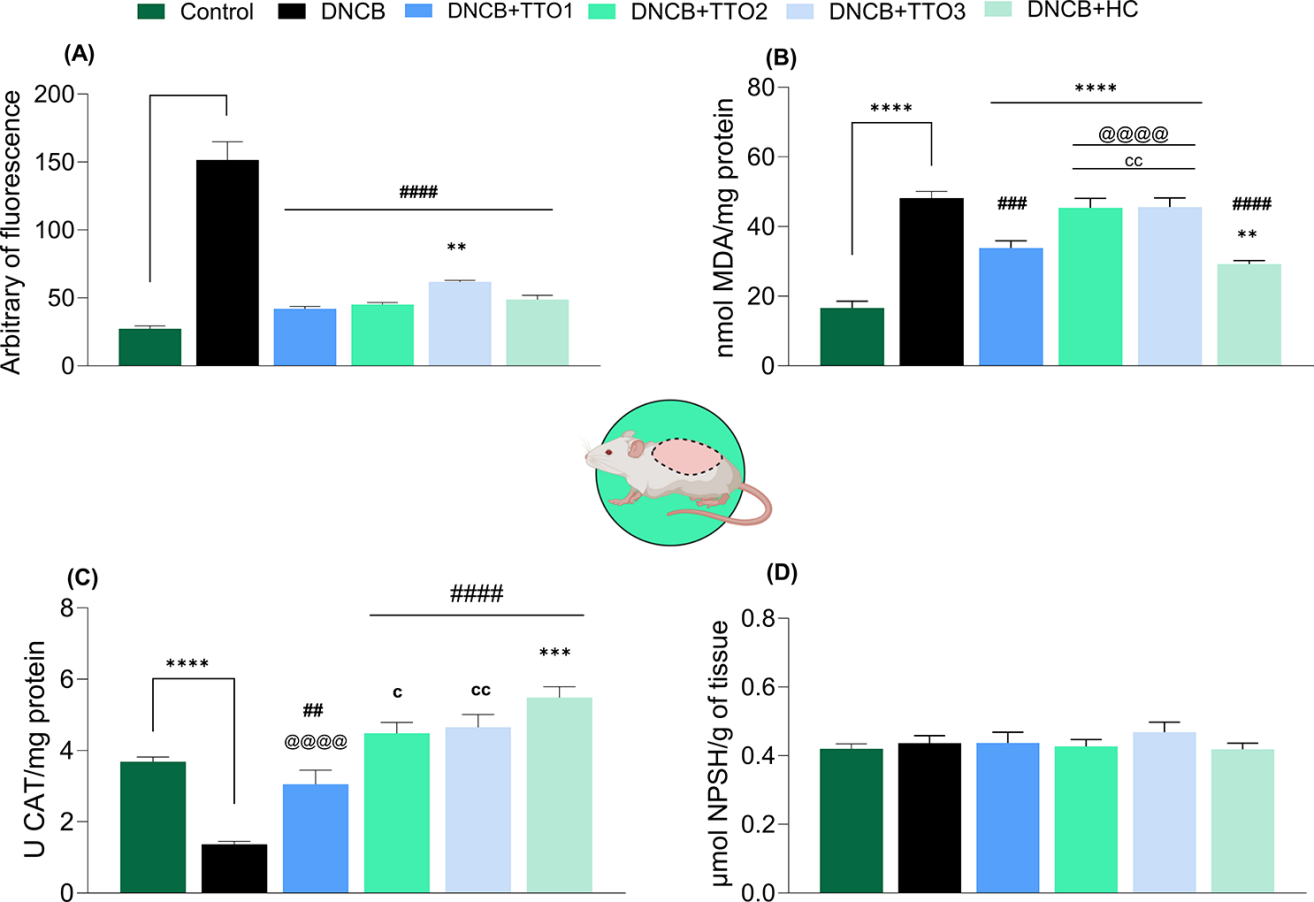
Effects of cutaneous administration of TTO1, TTO2, TTO3, and HC in the RS levels (A), TBARS levels (B), CAT activity (C), and NPSH levels (D) in the dorsal skin of mice. Data means ± SEM 7 animals per group (one-way ANOVA followed by Tukey’s test). (**) *p* < 0.01, (***) *p* < 0.001, and (****) *p* < 0.0001 compared with the control group; (##) *p* < 0.01, and (###) *p* < 0.001, and (####) *p* < 0.0001 compared with the DNCB group; (@@@) *p* < 0.001 and (@@@@) *p* < 0.0001 compared with the hydrocortisone (HC) group; (c) *p* < 0.05, (cc) *p* < 0.01 compared with the treatment 1 (TTO1) group.

In this context, endogenous antioxidant systems play a crucial role in attempting to restore redox balance in chronically inflamed tissues.^55^ These systems function by activating antioxidant enzymes, inducing cellular repair mechanisms, and reducing the production of RS. Here, we observed that DNCB exposure significantly inhibited the activity of the antioxidant enzyme CAT in the dorsal skin of mice compared with the control group (Figure 7C) (*F*_(5, 36)_ = 25.26, *p* < 0.0001).

This finding is consistent with previous studies indicating that excessive oxidative stress can compromise the structure and function of antioxidant enzymes, contributing to the progression of pro-oxidant states.^56^ Additionally, nonprotein thiol groups (NPSH), primarily represented by glutathione, have an essential role in maintaining the redox status of the organism.^57^ The reduction in NPSH levels is widely recognized as an indicator of oxidative damage. However, unlike the results observed for CAT, no significant differences in NPSH levels were identified between the experimental groups (Figure 7D) (*F*_(5,36)_ = 0.60, *p* > 0.05).

Daily administration of TTO1, TTO2, and TTO3 effectively modulated parameters associated with oxidative stress in the dorsal skin of DNCB-sensitized animals. The TTO1 reduced RS levels (Figure 7A) and lipid peroxidation (Figure 7B), as well as restoring CAT activity (Figure 7C). These results highlight its promising antioxidant properties and suggest therapeutic potential for redox imbalance conditions. Notably, its efficacy was comparable to that of HC, a reference glucocorticoid clinically used for AD management, as both promoted the recovery of the same oxidative parameters.

The TTO2 and TTO3 also demonstrated relevant antioxidant properties, promoting the reduction of RS levels and restoration of CAT activity. Although these treatments did not significantly affect lipid peroxidation, they contributed to mitigating key markers of oxidative stress. The observed antioxidant activity may be related to the intrinsic properties of the components used in the formulations. In the context, previous studies have reported that nanocellulose-based materials, particularly when combined with bioactive polysaccharides, can modulate oxidative processes by scavenging free radicals and protecting against lipid peroxidation.^58^ Similarly, mannan and its derivatives have demonstrated significant antioxidant capacity, including hydroxyl and superoxide radical scavenging, which may enhance tissue recovery and protection against oxidative damage.^21^ These findings suggest that the tested treatments, particularly TTO1, may offer promising therapeutic alternatives for oxidative stress and inflammation conditions, such as AD.

### 3.2 Evaluations of Therapeutic Effects of TTO1, TTO2, and TTO3 in the Diabetic Wound Healing

#### 3.2.1 The Administration of STZ Induces Phenotypic Diabetes in Mice

The administration of STZ is widely used to induce diabetes in animal models because it selectively injures pancreatic β cells, leading to persistent hyperglycemia. Due to its chemical similarity to glucose, STZ is rapidly taken up by the glucose transporter (GLUT2) found in β cells, which produce insulin. STZ-induced diabetic phenotype replicates conditions observed in diabetic patients, such as elevated blood glucose levels and impaired wound healing, making it a robust model for studying wound healing complications.^59^

As expected, on day 0 (ANOVA: *F*_(4,37)_ = 38.61, *p* <  0.0001) and day 12 (ANOVA: *F*_(4,37)_ = 145.5, *p* < 0.0001), the STZ administration induced an increase in the blood glucose levels when compared to the control group. The glucose levels measured on day 12 showed that daily sprayed administration of the treatments did not have an antihyperglycemic effect in diabetic mice (*p* > 0.05) (Table 2).

**Table 2:** Effect of STZ Administration in the Glucose Levels of Mice^a^

glucose levels (mg/dL)		
experimental groups	day 0	day 12
Control	125.1 ± 16.1	142.8 ± 9.2
STZ	280.3 ± 15.6****	356.6 ± 9.2****
STZ + TTO1	297.1 ± 10.1****	367.9 ± 5.7****
STZ + TTO2	287.6 ± 7.0****	395.7 ± 5.1****
STZ + TTO3	301.4 ± 7.9****	385.0 ± 16.3****

a Values are expressed as mean ± SEM of 7–10 mice per group. Data were evaluated through ordinary one-way ANOVA followed by Tukey’s test. (****) *p* < 0.0001 denotes significance levels when compared to the control group.

#### 3.2.2 Effect of Cutaneous Administrations of the TTO1, TTO2, and TTO3 in Wound Area Regression

Given that delayed wound healing is a common complication of *Diabetes mellitus*, the present study aimed to evaluate the therapeutic effect of TTO1, TTO2, and TTO3 in the diabetic wound model. The TTO1, TTO2, and TTO3 were applied daily to a 9 mm excisional wound in the dorsal back of mice. The wound area was measured on days 0 and 12 to determine the regression of the injured site (Figure 8A). The results revealed that diabetic mice have a poor wound-healing capacity compared with the control group. Analyzing the data on wound area closure revealed that only TTO2 cutaneous administration improved wound closure in diabetic mice when compared with the STZ group (Figure 8A,B) (ANOVA: *F*_(4,33)_ = 4.45, *p* < 0.01). No significant alteration was observed after the vehicle treatment in the wound area closure in nondiabetic mice (*p* > 0.05).

**Figure 8:**
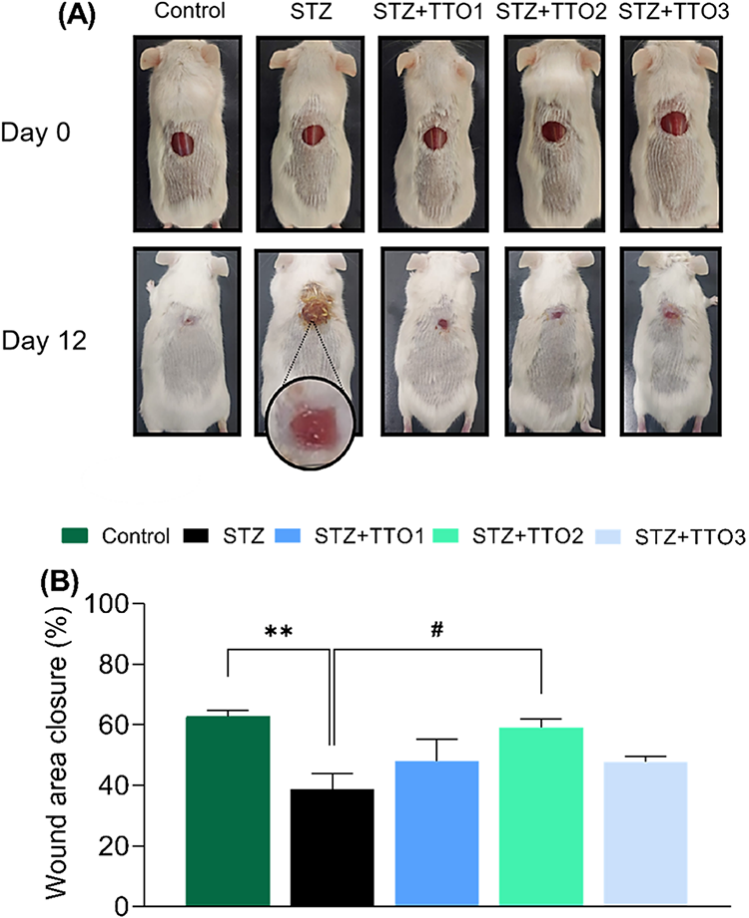
Effect of cutaneous administrations of TTO1, TTO2, and TTO3 in the wound area regression. (A) Representative photos of the extent of wound healing of one animal per group; (B) wound area closure (%). Data on wound area closure are reported as mean  ±  standard error of the mean (S.E.M.) of 7–10 animals per group (one-way analysis of variance/Tukey’s test). (**) *p* < 0.01 denotes significance levels when compared to the control group. (#) *p* < 0.05 denotes significance levels when compared to the STZ group.

#### 3.2.3 Effect of Cutaneous Administration of the TTO1, TTO2, and TTO3 in the Wound Bacteria Count

An intense inflammatory response in wounds can hinder the healing process. The accumulation of edema and excess fluid (exudate) creates an environment favorable for bacterial growth. This bacterial proliferation, in turn, inhibits the activity of fibroblasts, cells responsible for collagen production, which is essential for new tissue formation.^60^ In this context, the development of antibacterial dosage forms for skin wound treatment has gained increasing relevance, as these agents help prevent contamination and infection, allowing the healing process to proceed more efficiently.

Figure 9 illustrates the bacterial counts in the wound following cutaneous administration of TTO1, TTO2, and TTO3 (*F*_(4,19)_ = 6.88, *p* < 0.001). In diabetic animals, TTO2 significantly reduced CFU counts compared to the STZ group, indicating its promising antimicrobial potential. In contrast, TTO1 and TTO3 did not reduce CFU counts relative to the STZ group.

**Figure 9:**
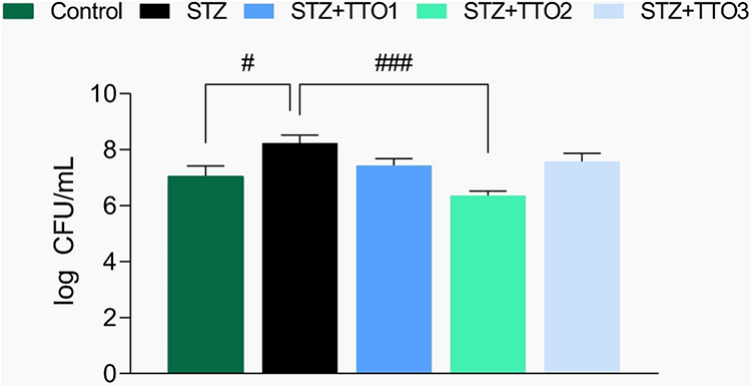
Number of CFU bacteria following cutaneous TTO1, TTO2, and TTO3 administration. The results were expressed in CFU/mL. Values are means ±  standard error of the mean (S.E.M.) of 5 animals per group (one-way analysis of variance/Tukey’s test). (#) *p* < 0.05, and (###) *p* < 0.001 denotes a significant result when compared to the vehicle (STZ) group.

#### 3.2.4 Effect of Cutaneous Administration of the TTO1, TTO2, and TTO3 in the Histological Changes

Figure 10 presents morphological alterations in the wound area of diabetic (STZ) and nondiabetic mice after cutaneous administrations of TTO1, TTO2, and TTO3. In the control group, nondiabetic and untreated, some ulcerated areas remained, but healed ulcers or ulcers in an advanced stage of healing predominated. The ulcerated areas showed dermal thickening, neovascularization, and mild inflammation (Figure 10A). In healed areas, acanthosis and evident collagen production were observed (Figure 10B,C).

**Figure 10:**
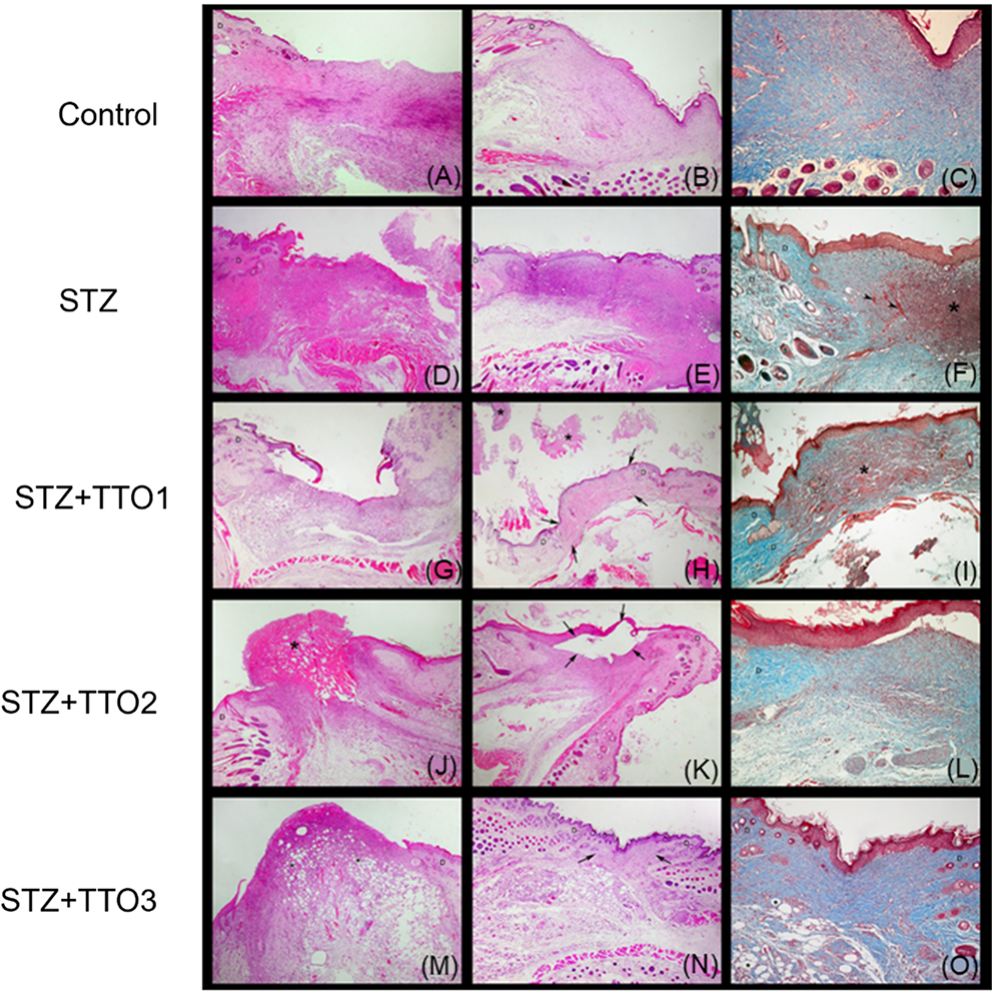
Histological profiles of the wound area in diabetic (STZ) mice after cutaneous administration of TTO1, TTO2, and TTO3. Control mice–(A) Ulcerated skin showing a homogeneous inflammatory response. Normal dermal region (D). HE. 100× objective lens; (B) Healed skin exhibiting acanthosis, dermal thickening, and mild inflammatory infiltrate (+) in the dermis and subcutaneous tissue. Normal dermal region (D). HE. 100× objective lens; (C) Healing area showing a good proportion of fibroblasts, collagen (+++), and a good supply of blood vessels. MT. 200× objective lens. Diabetic mice (STZ)–(D) Ulceration area with the inflammatory process extending to the subcutaneous tissue. Normal dermal region (D). HE. 100× objective lens; (E) Healing area showing acanthosis and marked (+++) inflammatory response in the dermis. Normal dermal region (D). HE. 100× objective lens; (F) Dermal region (*) exhibiting many fibroblasts but low collagen production. Blood vessel (arrowhead). Normal dermal region (D). MT. 200× objective lens. Diabetic mice (STZ) that received daily cutaneous administration of TTO1–(G) Ulcerated skin showed a mild inflammatory response. Normal dermal region (D). HE. 100× objective lens; (H) Healed skin exhibiting crusts (*). Scar area (between arrows); Normal dermal region (D). HE. 100× objective lens; (I) Healed skin where the dermis (*) exhibits a large amount of fibroblasts (reddish brown) interspersed with little collagen production (light blue). Normal dermal region (D). MT. 200× objective lens. Diabetic mice (STZ) that received daily cutaneous administration of TTO2–(J) Ulceration area exhibited an inflammatory response extending to the subcutaneous region. At the edge of the ulcer, a large amount of blood (*) is observed. Normal dermal region (D). HE. 100× objective lens; (K) Healed skin with a moderate inflammatory response in the dermis and subcutaneous tissue. Presence of a processing artifact with mechanical detachment of the epidermis (between arrows). Normal dermal region (D). HE. 100× objective lens; (L) Healed skin showing a homogeneous distribution of fibroblasts and collagen in the dermis. Normal dermal region (D). MT. 200× objective lens. Diabetic mice (STZ) that received daily cutaneous administration of TTO3–(M) Ulcerated skin showed a more controlled inflammatory response with a greater inflammatory infiltrate in the superficial region and many dilated lymphatic vessels (*). Normal dermal region (D). HE. 100× objective lens; (N) Healed skin exhibiting mild (+) inflammatory infiltrate in the dermis and subcutaneous tissue. Presence of dilated lymphatic vessels in the subcutaneous tissue. Scar area (between arrows). Normal dermal region (D). HE. 100× objective lens; (O) Healing area showing fibroblasts, good collagen production (+++), and good blood vessel supply. Presence of dilated lymphatic vessels (*) in the subcutaneous tissue. Normal dermal region (D). MT. 200× objective lens.

In diabetic (STZ group) mice, the dorsal skin presented ulcerated areas with an intense inflammatory process characterized by a massive infiltrate of neutrophils and, to a lesser extent, lymphocytes, and macrophages in the superficial and deep dermis and occasionally in the subcutaneous tissue (Figure 10D). Areas undergoing healing exhibited acanthosis and a marked inflammatory response in the dermis (Figure 10E) and subcutaneous tissue. Additionally, an extensive proliferation of fibroblasts was observed in the dermis, although collagen production in the healed areas was low (Figure 10F). In this group, ulcerated areas predominated over healed areas.

The diabetic mice that received cutaneous administration of TTO1 showed inflammatory response patterns. In some fragments, there was intense inflammation with a predominance of neutrophils, multiple purulent foci, and significant infiltration of eosinophils, macrophages, and lymphocytes in the superficial and deep dermis and subcutaneous tissue. In addition, dilated lymphatic vessels and congested blood vessels were observed to have a more pronounced infiltration of macrophages and lymphocytes than in the untreated STZ group. In other fragments, the inflammation was milder (Figure 10G), with ulcerated areas covered by crusts containing bacterial colonies (Figure 10H). Few fragments showed signs of healing. Trichrome staining revealed low collagen production at the edges of the ulcers and in the adjacent dermis, even in the only healed area (Figure 10I).

The ulcerated areas were predominated by an abundant inflammatory infiltrate of neutrophils and a discrete infiltrate of macrophages in the dermis and in extensive subcutaneous regions of the diabetic mice that received cutaneous administration of TTO2. Lymphatic vessels were dilated, and many newly formed blood vessels were found, with areas of hemorrhage visible at the edges of the ulcers (Figure 10J). Despite the predominance of the ulcerated regions, the inflammatory response was moderate (Figure 10K), and vascular proliferation was less intense. Trichrome staining revealed weak collagen production and little fibroblast proliferation compared to Figures 3 and 6. In healed fragments, moderate collagen production was observed (Figure 10L) and more significant vascular proliferation when compared to TTO1 and STZ groups.

Finally, the cutaneous application of TTO3 presented the best response to treatment, with most diabetic ulcers in the healing process. The inflammatory process was mild, and the healed areas exhibited acanthosis and dense collagen in the superficial and deep dermis (Figure 10N). In ulcerated areas, inflammation was more controlled, with a predominant infiltrate of macrophages and lymphocytes, less presence of neutrophils, and dilated lymphatic vessels (Figure 10M). Healed areas demonstrated an abundance of young fibroblasts, high collagen production, and good vascularization (Figure 10O). The inflammatory infiltrate was concentrated in the superficial and deep dermis and was rare in the subcutaneous tissue or adjacent musculature.

Histological analysis revealed distinct wound healing patterns in diabetic and nondiabetic mice. Diabetic animals exhibited more pronounced inflammation and impaired wound closure. The TTO3 administration demonstrated the most promising results: reduced inflammation, increased collagen deposition, and enhanced angiogenesis. This suggests a significant improvement in wound healing in diabetic mice.

In contrast, while TTO1 showed some beneficial effects, its response was more variable, with persistent inflammation and limited collagen production observed in some cases. The TTO2, although moderately reducing inflammation, exhibited limited collagen deposition, indicating a less favorable outcome than TTO3. These findings underscore the potential of targeted treatment modalities to modulate wound healing in diabetic mice.

### 3.3 Characterization of the Colloidal Suspensions

To better understand the biological results reported in the previous sections, we first characterized the structural and colloidal features of the synthesized nanocrystals and their suspensions. The CNC obtained by acid hydrolysis displayed an average width of 3.5 nm and a length ranging from 300 to 800 nm (TEM, Figure S1A,B), a hydrodynamic diameter of 398 nm (PDI ≈ 0.13, DLS), and a sulfation degree of 0.13 mmol/g. The MN exhibited a ribbon-like morphology with lengths ranging from 100 to 350 nm and an average diameter of approximately 82 nm (Figure S1C). These nanoscale dimensions and surface characteristics are consistent with those reported for cellulose- and mannan-derived nanocrystals prepared under comparable hydrolytic conditions, confirming that the adopted extraction route efficiently preserved the crystalline domains of both polysaccharides.^61,62^

Regarding coating efficiency, TTO1, using PMM-AC1 as the spray, exhibited the best performance, forming a continuous film after repeated application of this colloidal solution. As qualitatively demonstrated in Figure S2 (Supporting Information), only this colloidal suspension effectively coated the target area following the TTO1 protocol, whereas TTO2 and TTO3, which used different suspensions, showed limited capacity to form homogeneous and continuous coatings. These results can be attributed to the high viscosity of PMM-AC1 compared to other suspensions, which prevents rapid solvent evaporation. This facilitates the formation of intra- and intermolecular interactions between CNC and MN, thereby promoting film formation.^63^ Figures 11A–C present SEM images of coating fragments recovered from Petri dishes sprayed according to the different tested treatments. As observed, the composition of the colloidal suspensions had a significant impact on the microstructure of the coatings. In general, a reduction in the MN content within the suspensions led to decreased heterogeneity and roughness in the formed coatings, suggesting greater compatibility or enhanced interaction between CNC molecules, likely facilitated by the glycerol present in the formulation. Glycerol has been widely used as a compatibilizing agent in various films formulated or reinforced with CNC.^64^

**Figure 11:**
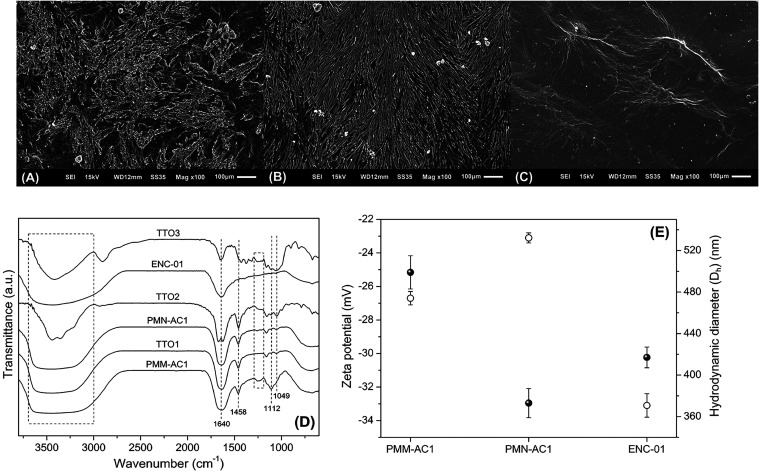
Images obtained by SEM from coatings formed by (A) TT01, (B) TT02, and (C) TTO3. (D) FTIR spectra recorded from the lyophilized colloidal suspensions (PMM-AC1, PMN-AC1, and ENC-01) and the coatings formed by TT01, TT02, and TTO3. (E) Zeta potential (hollow circles) and hydrodynamic diameters (*D*_h_) (filled circles) measured for PMM-AC1, PMN-AC1, and ENC-01.

The high coating efficiency demonstrated by TTO1 may explain its superior performance in treating and alleviating AD-like symptoms, as it enhances the physical barrier effect, preventing moisture loss and maintaining hydration in the treated area. These features are crucial for mitigating the main clinical conditions associated with AD.^65^ Furthermore, combining CNC and MN in an equal mass ratio may also contribute to this effect, as both components are hydrophilic, reinforcing the hydration properties of the formed coating. FTIR spectra recorded from PMM-AC1 and the resulting coating generated by TTO1 suggest a strong interaction between CNC and MN within this colloidal suspension. Figure 11D shows that the FTIR spectrum of PMM-AC1 exhibits the main bands corresponding to CNC and MN. For instance, the broad band in the range of 3700–3000 cm^–1^ corresponds to O–H stretching (hydroxyl groups), while the bands at 1640, 1458, 1112, and 1049 cm^1^ are attributed to O–H bending (adsorbed water), C–H bending (CH_2_ groups at the C6 position), and C–O–C and C–OH stretching, respectively.^62,66^ Due to their chemical similarity, the bands of CNC and MN appear in the same spectral range. However, the bands associated with asymmetric and symmetric S=O stretching (sulfated groups of CNC) are observable in the range of 1290–1180 cm^–1^.^61^ By comparing the FTIR spectra of PMM-AC1 and TTO1, a strong similarity can be observed, particularly in the range of 3700–3000 cm^–1^, where the broad band indicates extensive hydrogen bonding between CNC and MN. These strong intermolecular interactions likely promote partial aggregation of the nanocrystals within the colloidal suspension, which is consistent with the larger hydrodynamic diameter measured for PMM-AC1 (∼479 nm, Figure 11E). The corresponding particle size distribution curves obtained by DLS are provided in the Supporting Information (Figures S3a–c).

Further analysis of the FTIR data reveals that the spectra of the coatings formed by TTO2 and TTO3 differ from those of PMM-AC1 and ENC-01, likely due to reduced compatibility among the components in these suspensions. Overall, the spectra of TTO2 and TTO3 exhibited a narrowing of the band associated with O–H stretching (3700–3000 cm^–1^), indicating a reduction in inter- and intramolecular hydrogen bonding. This observation aligns with the lower film-forming capacity of the PMN-AC1 and ENC-01 suspensions. For PMM-AC1, the reduced compatibility between CNC and MN is reflected in the decrease in both the *D*_h_ of the colloids (∼373 nm) and their zeta potential (−23.1 mV) (Figure 11E). While the spectrum of TTO2 displayed additional bands attributed to the crystalline nature of MN at approximately 3440, 3350, and 3220 cm^–1^, the spectrum of TTO3 showed characteristic bands of CNC.^67^

The performance of the different treatments in the wound healing model differed from their effects on the AD model. In the wound healing model, neither the higher coating efficiency of TTO1 nor the increased MN content in the PMM-AC1 suspension resulted in the best biological responses in diabetic mice. Overall, TTO2 proved to be the most effective treatment for wound closure and bacterial reduction at the wound site, while TTO3 produced the best histological outcomes.

Regarding wound closure, the nonhomogeneous coatings formed by TTO2 and TTO3 likely facilitated the process by offering lower physical resistance to tissue regeneration compared to TTO1. Additionally, the reduced interaction between CNC and MN allowed each component to contribute to the healing process independently, with CNC appearing to play a dominant role. According to the literature, CNC-based biomaterials have been shown to enhance wound closure, promote re-epithelialization, and stimulate collagen deposition.^68,69^ On the other hand, the higher availability of MN in TTO2 contributed to bacterial colony control at the wound sites in diabetic mice. Previous studies suggest that mannan-based prebiotics can modulate the growth rate of antibiotic-resistant bacterial strains, such as *Escherichia coli*.^70^ In line with this, the lower negative zeta potential measured for PMM-AC1 could be advantageous, as bacterial surfaces typically carry a negative charge.

By integrating the biological outcomes from the tested models with the physicochemical characterization data, it becomes clear that the composition of the colloidal suspension is a key determinant of the observed therapeutic effects. Notably, the presence of MN was critical for treatment efficacy in most models, although its optimal concentration appears to be application-specific and warrants careful adjustment. Our findings support the initial hypothesis that colloidal suspensions comprising CNC and MN nanocrystals derived from ivory nuts represent promising sprayable biomaterials. These formulations demonstrate at least two major therapeutic functions: mitigating chronic skin conditions, such as atopic dermatitis, and enhancing wound healing in diabetic models. Future investigations should address current limitations and expand the range of potential clinical applications.

## 4 Conclusion

The colloidal suspensions of CNC and MN derived from ivory nut endosperms offer a promising alternative for treating atopic dermatitis (AD) and diabetic wound healing. The combined effects of these nanocrystals have demonstrated synergistic antimicrobial and anti-inflammatory properties. A sprayable colloidal suspension containing a 1:1 (w/w) ratio of CNC and MN exhibited the best results for alleviating AD symptoms. The high viscosity and strong interaction between CNC and MN in this formulation facilitated the formation of homogeneous coatings that maintained hydration at the treatment site, promoting the healing process. Conversely, a colloidal suspension with a lower MN content (6:1 w/w CNC/MN ratio) showed superior efficacy in wound healing in diabetic mice. In this composition, the reduced interaction between CNC and MN allowed each component to contribute more independently to the healing process. These findings suggest that the tested colloidal suspensions hold significant potential as sprayable biomaterials for treating AD and wounds, offering a sustainable alternative based on compounds derived from biomass. Additionally, their ease of application and reduced frequency of administration present notable advantages for patient comfort and adherence to treatment.

## Supplementary Material



## Data Availability

Data will be made available on request.

## Notes

Dynamic light scattering and zeta potential measurements; TEM and SEM images; FTIR spectra; photographic images of spray application areas (PDF)
