# Supplementary material for: Sprayable Cellulose and Mannan Nanocrystals from Ivory Nuts for Treatment of Skin Diseases in Mice
Source: ACS Appl Bio Mater. 2025 Nov 21;8(12):11019–35. doi: 10.1021/acsabm.5c01708 (PMC12709580; doi:10.1021/acsabm.5c01708)
Supplement: Supplementary file 1 [file mt5c01708_si_001.pdf]

Supporting information for

Sprayable Cellulose and Mannan Nanocrystals from Ivory Nuts for Treatment of Skin Diseases in Mice

Vanessa M. E. da Rocha[a], Ana Paula B. Wille[a], Ana Paula S. e Silva[a], Matheus S. Gularte[b], Mauro P. Soares[c], Marcelle M. Silveira[d], Janice Giongo[d, e], Rodrigo A. Vaucher[d], Jeff R. Spitzner[g], André R. Fajardo[b], Enrique Javier Carvajal-Barriga[f,g*], and Ethel A. Wilhelm[a*]

[a] *Preclinical and Translational Research Group in Pain and Chronic Diseases, Federal University of Pelotas (UFPel), 96010-900, Pelotas, RS, Brazil.*

[b] *Laboratory of Technology and Development of Composites and Polymer Materials (LaCoPol), Federal University of Pelotas (UFPel), 96010-900, Pelotas, RS, Brazil.*

[c] *Regional Diagnostic Laboratory Faculty of Veterinary Medicine, Federal University of Pelotas (UFPel), 96010-900, Pelotas, RS, Brazil.*

[d] *Laboratory of Biochemistry Research and Molecular Biology of Microorganisms (LaPeBBioM), Federal University of Pelotas (UFPel), 96010-900, Pelotas, RS, Brazil.*

[e] *Faculty of Medicine, Federal University of Rio Grande, 96203-900, Rio Grande, RS, Brazil.*

[f] *Neotropical Center for the Biomass Research (CNIB), Pontificia Universidad Católica del Ecuador (PUCE), Av. 12 de Octubre 1076 y Roca, Quito, Ecuador.*

[g] *PanoMatrix, LLC, 1476 Manning Pkwy, Powell, OH 43065 USA.*

(*) Corresponding authors:

Prof. E. A. Wilhelm, ORCID 0000-0002-2875-9962, e-mail: ethelwilhelm@yahoo.com.br; ethel.wilhelm@ufpel.edu.br - Phone: +55 53 32757360.

Prof. E.J. Carvajal, ORCID 0000-0002-0647-9513, e-mail: ejcarvajal@puce.edu.ec, Phone: +593 2 2991700

1. Experimental

1.1. Dynamic light scattering (DLS) and zeta potential measurements

The hydrodynamic diameter (D_h) of the particles in the colloidal suspensions described in Table 1 was evaluated using an Anton Paar Litesizer 500 (Austria) laser light scattering device, operating at a fixed angle of 90° and a wavelength of 658 nm, from a single-frequency laser diode providing 40 mW. All measurements were performed at 25 °C, neutral pH, in water. The diffusion coefficient values were obtained from the autocorrelation functions and correlated with the Stokes-Einstein equation, as the equipment software provided.

The zeta potential values were measured using the same Litesizer 500 equipment with an Omega cuvette (Mat. No. 225288) at neutral pH and 25 °C. The Smoluchowski approximation was used to convert the electrophoretic mobility into zeta potential values.[1] Diluted solutions were prepared in triplicate using the colloidal suspensions at 1 mg/mL concentration. All samples were stabilized inside the equipment for 30 s before measurement.

1.2. Transmission electron microscopy (TEM) and scanning electron microscopy (SEM)

Diluted aqueous suspensions of CNC and MN, dispersed using a probe ultrasound device, were deposited onto glow-discharged, carbon-coated TEM grids. Before drying, the samples were negatively stained with 2% uranyl acetate. The specimens were examined using a FEI Tecnai G2 Spirit BioTWIN microscope (USA) operating at 80 kV.

SEM images were used to investigate the morphology and microstructure of the coatings formed by the tested colloidal suspensions applied to the treated regions of the mice. To mimic the *in vivo* assay process, the colloidal suspensions (ENC-1, PMM-AC1, and PMN-AC1) were sprayed onto a designated area (1.0 cm in diameter) marked on a Petri dish, following the treatment protocol for the wound healing model (once a day for 12 days). The spray distance from the Petri dish was set to 5 cm, and after application, the dish was left under open-atmosphere conditions at room temperature. After the final day, the film-like coatings deposited on the sprayed areas were carefully recovered and mounted on aluminum stubs using a carbon adhesive and gold coated by sputtering. SEM images were recorded using a JEOL JSM-6610LV scanning electron microscope (USA).

1.3. Fourier transform infrared spectroscopy (FTIR)

The coating samples recovered from the Petri dishes were also analyzed using Fourier transform infrared (FTIR) spectroscopy. Spectra were recorded with a Shimadzu IR Affinity 1 spectrometer (Japan) operating within a spectral range of 4000–400 cm^{-1}, with a resolution of 4 cm^{-1} and 64 scans. Before recording the spectra, the samples were ground, mixed with KBr, and pressed into disks. FTIR spectra were also obtained from the colloidal suspensions (described in Table 1). To achieve this, the suspensions were frozen using liquid N_2 and subsequently lyophilized at -55 ºC for 24 h. The resulting powdered materials were then mixed with KBr, pressed into disks, and analyzed under the same operational conditions previously described.

2. Supplementary figures

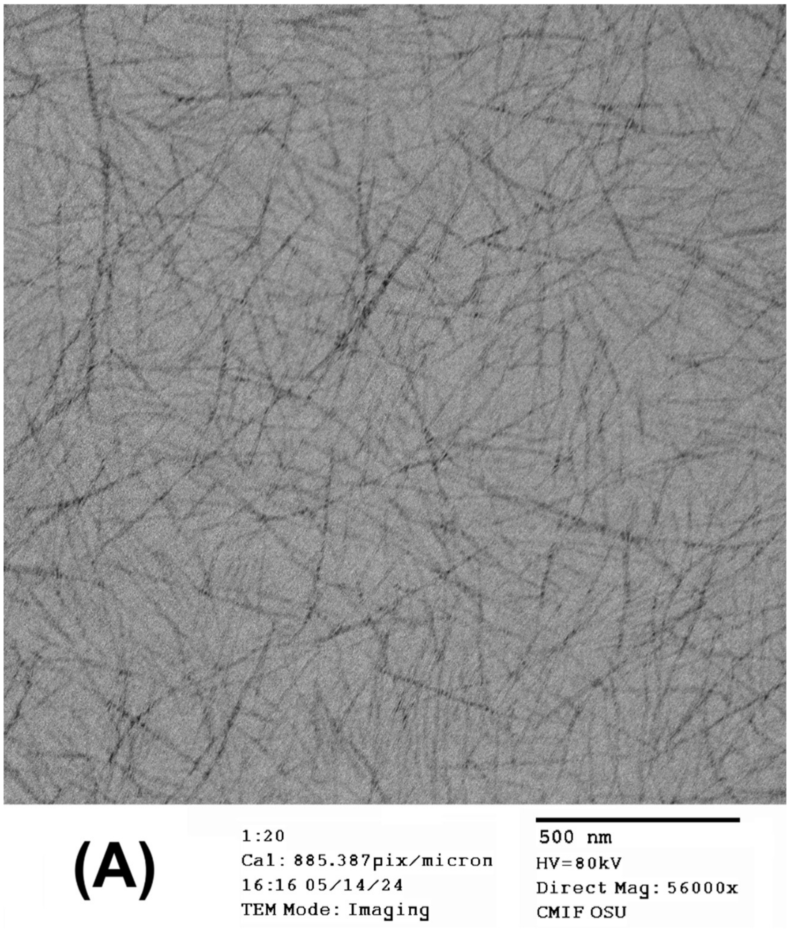

(A)

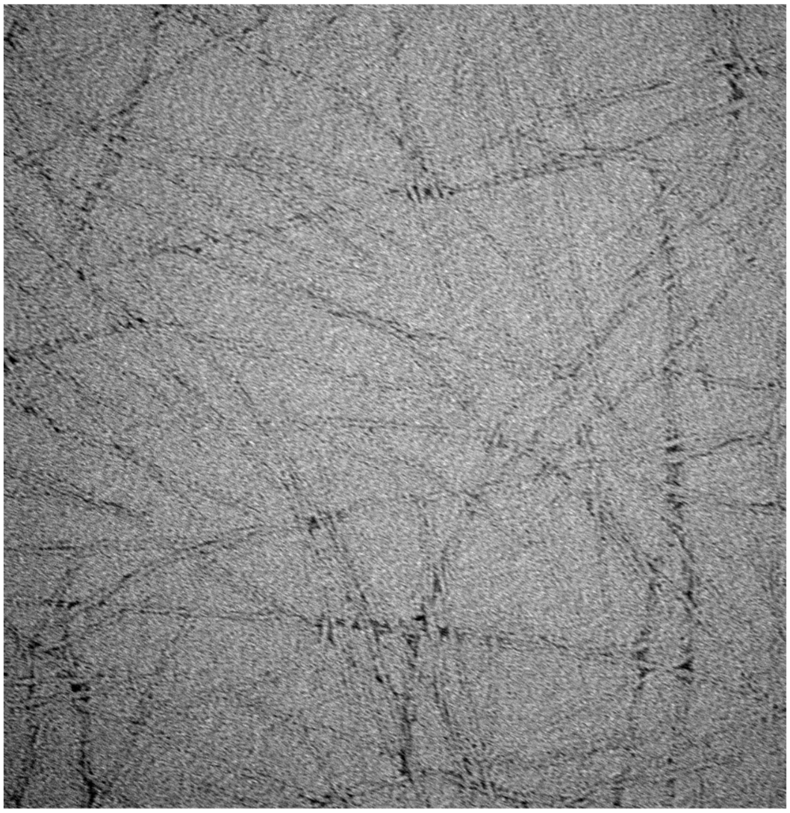
1:20
Cal: 2.76pix/nm
16:20 05/14/24
TEM Mode: Imaging
100 nm
HV=80kV
Direct Mag: 170000x
CMIF OSU

(B)

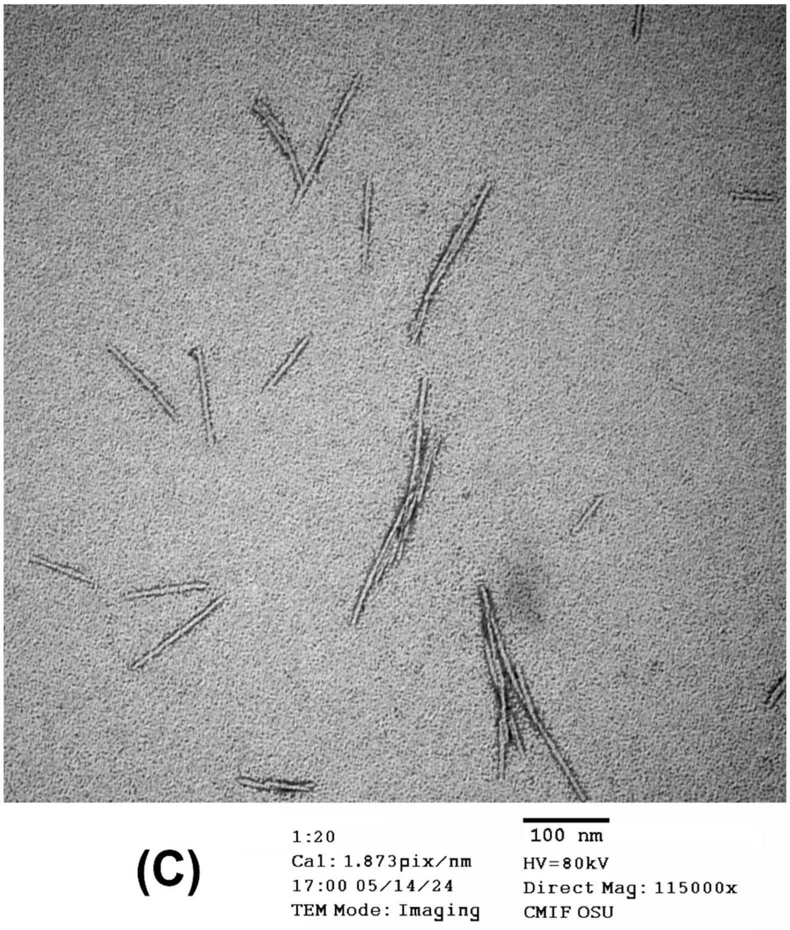

Figure S1. TEM images of (A,B) cellulose nanocrystals (CNC) at various magnifications and (C) mannan nanocrystals (MN).

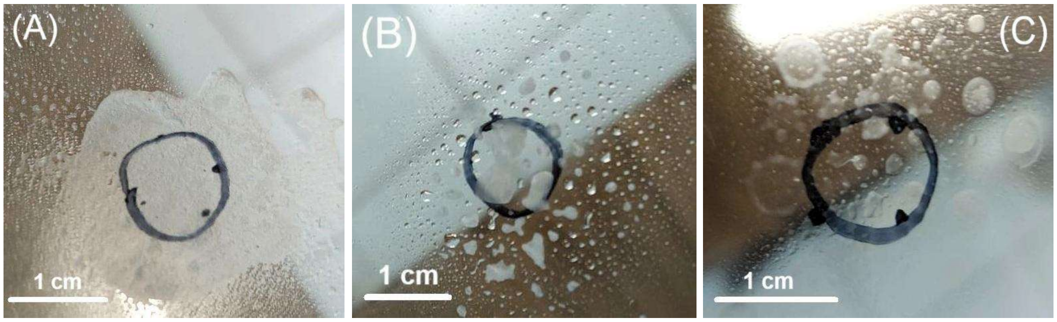

Figure S2. Photographic images of Petri dishes showing the application areas treated with (A) TTO1, (B) TTO2, and (C) TTO3 after 12 days of application. Each area was sprayed once per day.

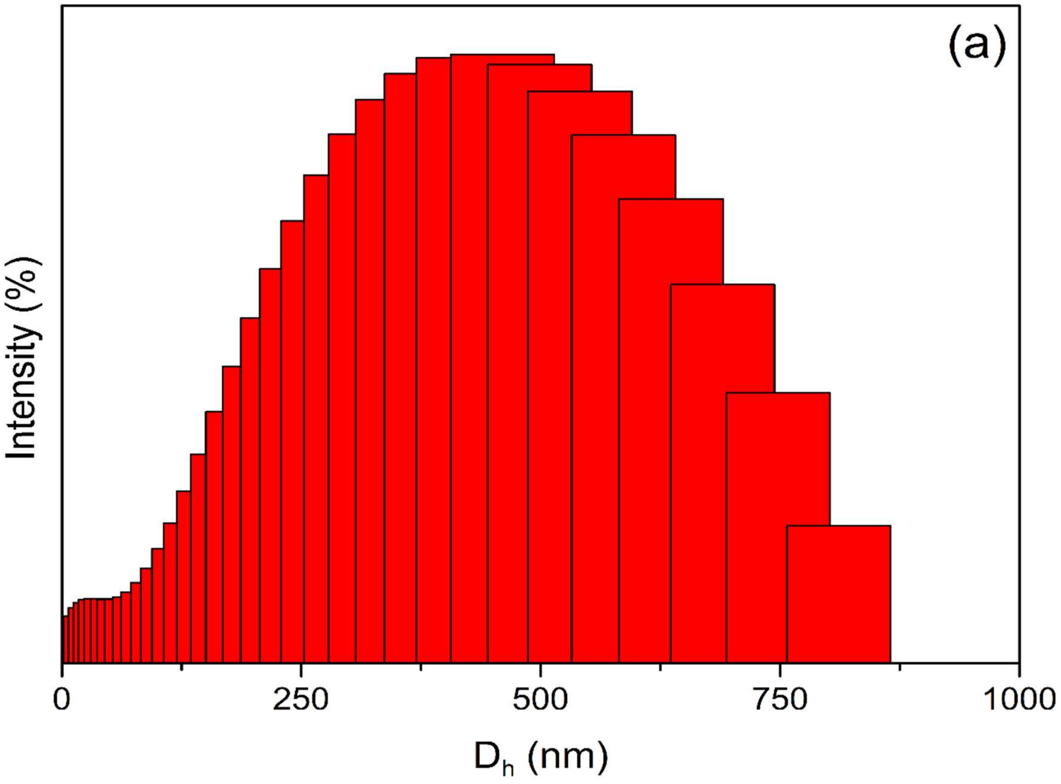
(a)
Intensity (%)
0
250
500
750
1000
D$_h$ (nm)

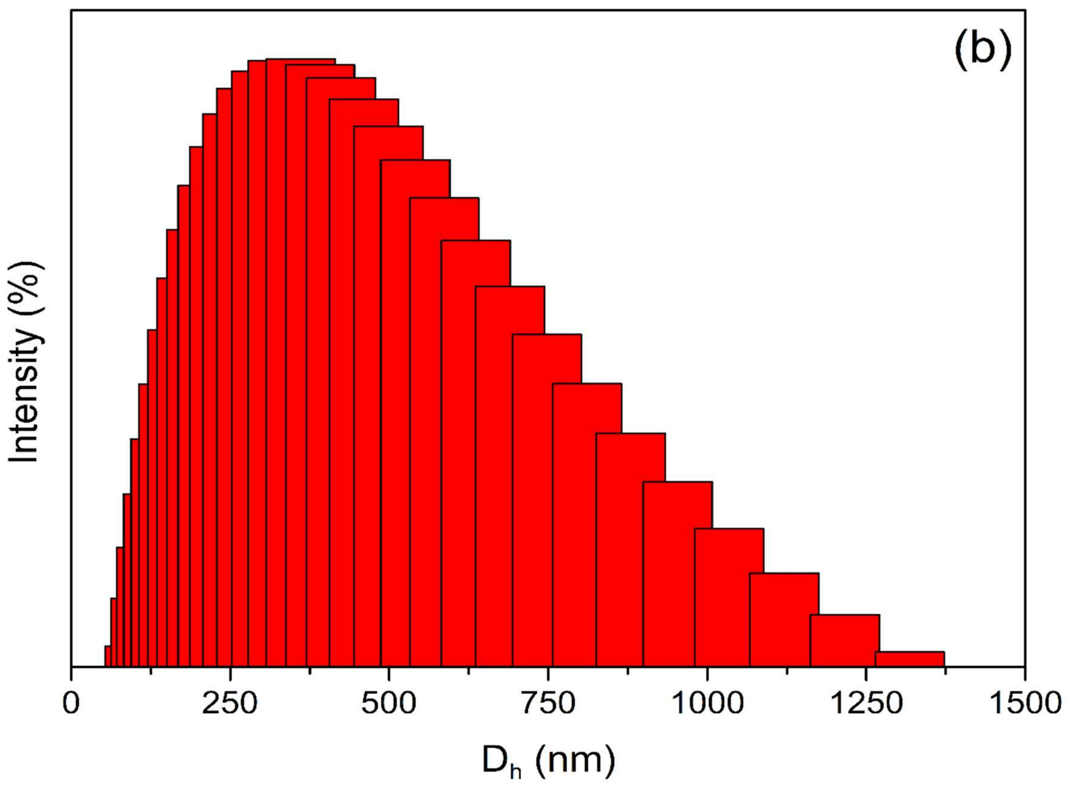

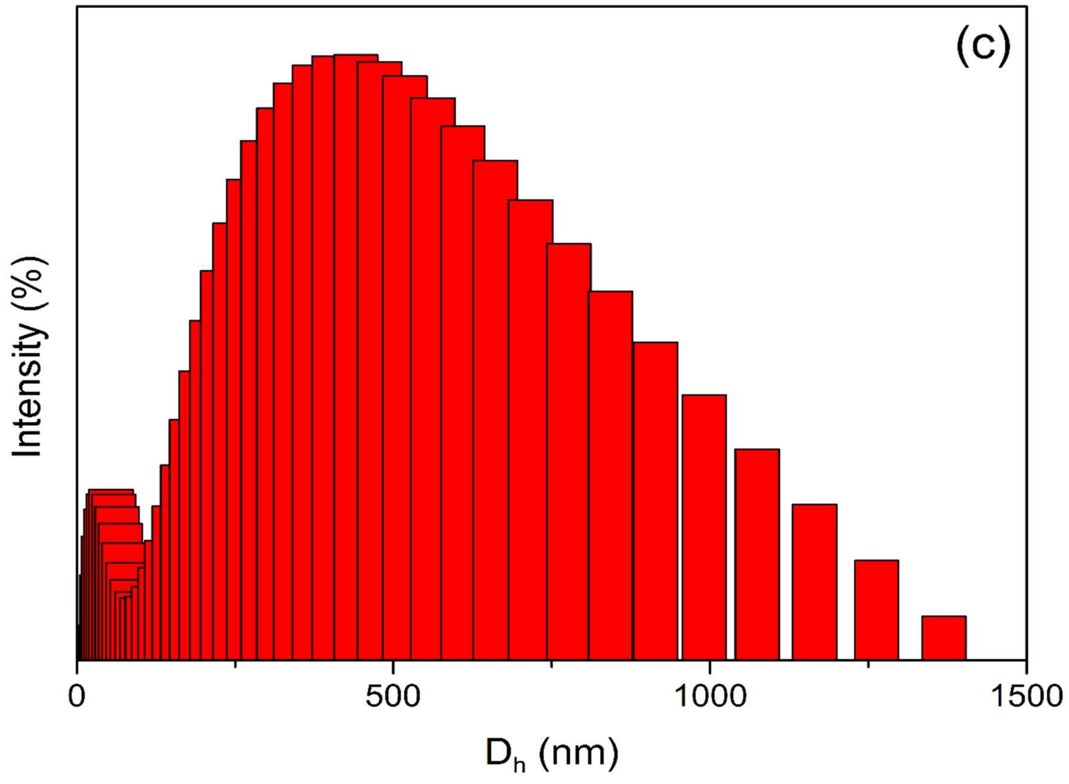

Figure S3. DLS intensity hydrodynamic diameter (D_h) distribution histograms for (a) PMM-AC1, (b) PMN-AC1, and (c) ENC-01 colloidal suspensions.

References

(1) Sze, A.; Erickson, D.; Ren, L.; Li, D. Zeta-Potential Measurement Using the Smoluchowski Equation and the Slope of the Current–Time Relationship in Electroosmotic Flow. *J. Colloid Interface Sci.* **2003**, *261* (2), 402–410. https://doi.org/10.1016/S0021-9797(03)00142-5.
